# Ecological restoration of agricultural land can improve its contribution to economic development

**DOI:** 10.1371/journal.pone.0247850

**Published:** 2021-03-05

**Authors:** Adrian C. Newton, Paul M. Evans, Stephen C. L. Watson, Lucy E. Ridding, Steven Brand, Morag McCracken, Arjan S. Gosal, James. M. Bullock

**Affiliations:** 1 Faculty of Science and Technology, Centre for Ecology, Environment and Sustainability, Bournemouth University, Poole, United Kingdom; 2 UK Centre for Ecology and Hydrology, Wallingford, United Kingdom; 3 Plymouth Business School, University of Plymouth, Plymouth, United Kingdom; Gebze Teknik Universitesi, TURKEY

## Abstract

Given the negative environmental impacts of intensive agriculture, there is an urgent need to reduce the impact of food production on biodiversity. Ecological restoration of farmland could potentially contribute to this goal. While the positive impacts of ecological restoration on biodiversity are well established, less evidence is available regarding impacts on economic development and employment. Potentially, prospects for economic development could be enhanced by ecological restoration though increased provision of ecosystem services, on which some economic activity depends. Here we examined this issue through the development of contrasting land use scenarios for the county of Dorset, southern England. Two scenarios of future agricultural expansion were compared with two scenarios of landscape-scale ecological restoration and the current situation. Impacts on provision of multiple ecosystem services (ES) were explored using InVEST models and proxy values for different land cover types. Impacts on economic employment were examined using an economic input-output model, which was adjusted for variation in ES flows using empirically determined ES dependency values for different economic sectors. Using the unadjusted input-output model, the scenarios had only a slight economic impact (≤ 0.3% Gross Value Added, GVA). Conversely, when the input-output model was adjusted to take account of ES flows, GVA increased by up to 5.4% in the restoration scenarios, whereas under the scenario with greatest agricultural expansion, GVA was reduced by -4.5%. Similarly, employment increased by up to 6.7% following restoration, compared to declines of up to -5.6% following maximum agricultural expansion. These results show that the economic contribution of rural land is far greater than that attributable to agricultural production alone. Landscape-scale restoration of agricultural land can potentially increase the contribution of farmland to economic development and employment, by increasing flows of multiple ES to the many economic sectors that depend on them.

## 1. Introduction

Approximately 40% of the Earth’s land surface is now being used for food production, indicating that agriculture is now the predominant type of land use [1]. The intensification and expansion of agriculture is widely being pursued as a policy option, with the aim of improving food security and supporting human development [2]. However, it is widely recognised that farming is often associated with a wide range of negative environmental impacts, which can include increased emissions of greenhouse gases (GHG) and ammonia; eutrophication; application of toxic herbicides and pesticides; depletion of freshwater; and increased soil compaction, depletion and erosion [3–5]. In particular, agriculture is recognised as the leading contributor to biodiversity loss, which principally occurs through the conversion of natural habitats to farmed systems. Consequently there is an urgent need to reduce the impact of food production on biodiversity, which could potentially be achieved by changing patterns of both food production and consumption, and through a combination of conservation, sustainable management and ecological restoration [6]. While many techniques and strategies are already available that can contribute to this goal, there is a particular need to scale them up from local to landscape or regional scales [6].

Ecological restoration of farmland has generally focused on moderating the effects of habitat fragmentation and loss, and reducing the intensity of management [7]. For example, Rey Benayas et al. [8,9] describe the establishment of wooded islets on farmland as a way of increasing connectivity of woodland habitats in agricultural landscapes. More widely, habitat improvements have been supported by the introduction of different types of agri-environmental scheme, such as those supported by the European Union (EU) Common Agricultural Policy (CAP) in more than 26 countries, and the Conservation Reserve Program in the USA [7]. Such approaches may directly support large-scale ecological restoration, or can usefully be combined with it [10]. The need to plan and implement restoration at relatively large spatial scales is supported by empirical evidence indicating that landscape-scale factors influence the abundance of key functional groups of species in agricultural landscapes, such as pollinators, seed dispersers and natural enemies [5,7,11].

While ecological restoration is often implemented through active management intervention, passive habitat recovery can also occur if farming is abandoned. Such abandonment is becoming increasingly widespread in many high-income countries, especially in small and extensive farming systems that are economically marginalised, for example those located in mountain areas [12]. The potential impact of land abandonment on biodiversity has been the focus of scientific debate. Many traditional, low-intensity farming landscapes can be highly species rich; examples include semi-natural grasslands in Europe, the *milpa* systems in South America and *satoyama* landscapes in Japan [13]. A number of investigations have demonstrated biodiversity declines in such landscapes following abandonment of agricultural practices [13]. Conversely, abandonment can provide an opportunity for the passive recovery of native ecosystems with high conservation value, for example forests or grasslands. This has stimulated interest in rewilding as a land use option [14]. For example in Europe, Navarro and Pereira [15] argue that traditional agriculture practices are not environmentally friendly, whereas rewilding involving passive management approaches could benefit a wide range of species, including 60 species of birds and 24 species of mammals. Although rewilding is increasingly being adopted as a land management approach, it is still controversial, partly owing to uncertainty regarding its ultimate outcomes [16–18].

The positive impacts of ecological restoration on biodiversity are now well established, as revealed by global meta-analyses. For example, in a meta-analysis of 89 restoration assessments undertaken in a wide range of different ecosystem types, Rey Benayas et al. [19] reported a 44% increase in biodiversity measures following restoration. Similarly, in a meta-analysis of 221 study landscapes worldwide, Crouzeilles et al. [20] found that forest restoration enhanced biodiversity by 15–84% and vegetation structure by 36–77%. A further analysis of 133 studies in tropical forests showed that natural regeneration provided significantly higher benefits for biodiversity (plants, birds and invertebrates) and vegetation structure than did active restoration approaches [21], strengthening the case for restoration using passive ecological recovery. With respect to agricultural ecosystems, Barral et al. [22] examined the results of 54 studies drawn from 20 countries, which showed an increase biodiversity measures following restoration by a mean of 68%.

However, less information is available regarding the potential impact of landscape-scale ecological restoration on human communities. In this context, the concept of ecosystem services (ES), or the benefits provided by ecosystems to people, is of particular value. According to analytical frameworks that are now widely being used to influence environmental policy, ecological restoration represents a form of investment in natural capital, which could benefit human well-being by increasing ES flows to people [23]. A number of studies have provided evidence that ecological restoration can increase ES flows; for example, in their global meta-analysis, Rey Benayas et al. [19] found that that restoration increased provision of ES by 25%, although values tended to remain lower in restored than in intact reference ecosystems. Similarly, in their review of agricultural systems, Barral et al. [22] reported that provision of supporting and regulating ES increased by means of 42% and 120% respectively, relative to values recorded prior to restoration. Previous research has also documented projected increases in ES provision when ecological restoration is undertaken at the landscape scale (e.g. [24,25]). However, few studies have explicitly examined rewilding in this context (e.g. see [24]); the evidence summarized by Cerqueira et al. [26] is based on analysis of wilderness areas as a proxy for future rewilding, rather than the outcomes of actual rewilding initiatives.

In recent years, considerable progress has been made in assessing ES values in different contexts, and in understanding how these values inform decision-making relating to land use [27]. However, there are important gaps in the research undertaken to date. For example, comprehensive ES analyses should assess both the supply of and demand for ES, but the vast majority of studies have examined only the supply side [28]. Demands for ES can be evaluated using economic valuation techniques in real or hypothetical markets, or by assessing people’s perceptions of the importance of different ES, yet in practice this is done relatively rarely [28]. Furthermore, most studies only provide a static analysis of the current situation, and fail to consider how ES flows might change over time, despite the importance of providing forecasts to inform policy and land-use decisions. Although forecasts of ES dynamics can be developed through the use of scenario approaches [28], these have not been widely applied to date. Consequently, there is a lack of evidence regarding the changes in ES flows that might arise as a result of ecological restoration, and how these relate to economic demands.

Here we explore the potential impacts of landscape-scale ecological restoration on natural capital, and associated provision of ES, in a lowland agricultural landscape in southern England. The UK currently provides a valuable opportunity to examine this issue, given its departure from the European Union (EU) and the EU’s Common Agricultural Policy (CAP) schemes, which have formerly been an important source of revenue for landowners. As a result of Brexit, the UK is currently in the process of developing new land use policies, which may enable landowners to receive government funds for the provision of ES [29]. This represents a profound policy shift that might provide economic opportunities for large-scale ecological restoration and rewilding. Potentially, such interventions could provide benefits to economic development and employment, if they increase provision of those ES that support economic activity. Here we examine this possibility through the use of scenarios of potential future land use options, including both landscape-scale restoration and agricultural expansion, which we compare with the current situation. Using models and proxies of ES provision, we forecast projected changes in ES delivery over an interval of 35 years under these different scenarios. The potential impacts on economic growth and employment are then explored using an economic input-output model, an approach that is widely used to inform regional development planning [30]. Specifically, this research was designed to test the following hypothesis: landscape-scale ecological restoration of agricultural land will increase its contribution to economic development, by increasing the provision of multiple ES for which there is economic demand.

## 2. Materials and methods

### 2.1. Study area and land cover maps

The county of Dorset is situated in southern England. The current landscape is predominantly agricultural, comprising 32% arable cropland and 48% improved grassland by area. Other land cover types include urban (6%), native broadleaved woodland (6%), conifer plantations (3%), lowland heathland (2%) and unimproved grassland (including calcareous grassland)(1%). These land cover values are broadly typical of other lowland rural areas of north-west Europe. This specific area was selected for study owing to the unusual availability of time-series data describing both biodiversity and land cover, spanning more than 80 years. To provide a baseline for assessing the impacts of land use changes on natural capital, we used a land cover map for 1930 produced by Hooftman and Bullock [31] based on a survey undertaken in the 1930s [32]. In addition, a 2015 map was derived from the CEH Land Cover Map 2015 (LCM2015) [33]. Details of how these maps were produced are provided by Ridding et al. [34–36]. Although Dorset is currently ca. 2653 km^2^ in area, its boundaries were extended in 1974 to include the towns of Bournemouth, Poole and Christchurch; prior to this date, the area of the county was ca. 2500 km^2^ [31]. We employed the latter boundary for the current study, to enable changes over time to be assessed. All maps were produced at a resolution of 100 m x 100 m, to ensure consistency across all of the data sets, and processed using ArcGIS v 10.1 (ESRI, Redlands, California, USA).

### 2.2. Scenarios of land cover change

To explore the potential impacts of future land cover change, we developed five scenarios for the period 2015–2050, which were designed to cover a wide spectrum of possibilities: (i) “Business as usual” (BAU), in which the land cover of Dorset remains unchanged; (ii) “High Intensity Green Brexit” (HIGB), in which the area of agricultural land (i.e. arable cropland plus improved grassland) declines by 41.5% over the 35 year interval; (iii) “Low Intensity Green Brexit” (LIGB), in which the area of agricultural land declines by 20.7%; (iv) “Low Intensity Agribrexit” (LIAB), in which the area of agricultural land increases by 9%; and (v) “High Intensity Agribrexit” (HIAB), in which the area of agricultural land increases by 18%, relative to the BAU value (Table 1). The 35 year duration of the scenarios was designed to be relevant to the timescale of current strategic planning within the study area.

**Table 1:** The extent of land cover change in the different scenarios.

Land cover type	BAU (%)	HIGB (%)	LIGB (%)	HIAB (%)	LIAB (%)
Acid grassland	0.15	0.15	0.15	0	0
Arable cropland	30.0	19.8	24	32.3	31.7
Broadleaved, mixed and yew woodland	6.24	20	13.9	0	3.11
Built-up areas and gardens	8.14	8.14	8.14	8.14	8.14
Calcareous grassland	0.6	10.4	6.12	0	0
Coastal	1.68	1.68	1.68	1.68	1.68
Coniferous woodland	3.66	3.66	3.66	0	3.66
Fen, Marsh, Swamp (incl. Saltmarsh)	0.53	2.32	0.85	0.53	0.53
Heathland	2.64	5.18	3.27	0	0
Improved grassland	45.5	24.4	35.8	56.7	50.6
Inland rock	0.31	0.37	0.32	0.31	0.31
Inland water	0.28	0.28	0.28	0.28	0.28
Neutral grassland	0.28	3.64	1.74	0	0

Values presented are percentages of total land cover. BAU: “Business as usual”; HIGB “Green Brexit”, High Intensity; LIGB “Green Brexit”, Low Intensity; HIAB “Agribrexit”, High Intensity; LIAB “Agribrexit”, Low Intensity.

Land cover maps were produced for each of the scenarios based on LCM2015 [33]. For the BAU scenario, representing no future land cover change, LCM2015 was used in an unaltered form. To produce the land cover maps for ecological restoration (LIGB and HIGB), we used the South-West Nature Map (http://www.biodiversitysouthwest.org.uk/nm_dwd.html). This represents a regional approach to landscape-scale planning for habitat restoration that was developed by conservation organisations in South West England. Areas proposed for restoration of priority habitats are referred to on the map as Strategic Nature Areas (SNAs), and were identified using a combination of research and expert judgement following an ecoregional planning approach [37,38]. The land cover map for HIGB was based on complete implementation of the Nature Map, by converting all pixels of agricultural land that were overlayed by SNAs to their respective semi-natural habitat type. The LIGB land cover map was produced by halving the number of pixels of agricultural land that were converted in this way; the pixels were selected by using a buffering procedure within ArcGIS. LIGB therefore represents 50% implementation of the SNAs.

For the “Agribrexit” scenarios (HIAB and LIAB), remaining semi-natural habitats that are suitable for agriculture were converted to farmland; the type of agriculture in each location (i.e. arable cropland or livestock) was determined by the relative suitability of different soil types. For this purpose, soil data were obtained from (NATMAP National Soil Map; National Soil Resources Institute, Silsoe, Bedfordshire, UK). Specifically sandy, clay and peaty soils became improved grassland, whereas lime-rich and high fertility loams were converted to arable cropland. In the HIAB scenario, all of the following habitats were converted to agriculture: neutral, calcareous and acid grassland; heathland; broadleaved and coniferous woodland. The total increase in agricultural land of 18% therefore represents the maximum amount of conversion to farmland that is possible in this study area. The LIAB implemented half of this value of land cover change (i.e. 9%). Selection of pixels for conversion under this scenario was conducted using a buffering procedure using the LCM2015 map, in which areas of new agricultural land were situated adjacent to existing farmland. The buffer distance was increased until the target of 9% expansion of agricultural land was reached.

### 2.3. Habitat fragmentation analysis

To assess the impact of land cover change on habitat fragmentation, the land cover maps associated with each scenario were analysed using FRAGSTATS (v4) [39]. We used the following class metrics generated by FRAGSTATS: Patch Density, Mean Patch Size, Edge Density (using a value of 100 m edge depth), Mean Shape Index, Mean Core Area, Euclidean nearest-neighbour distance and Interspersion and Juxtaposition Index. Values of each of these metrics were calculated for each land cover type in each scenario, as well as the 1930 land cover map.

### 2.4. Ecosystem service assessment

The InVEST suite of models has been widely used to examine the spatial dynamics of ecosystem service (ES) flows in different contexts [40,41]. The models are based on production functions relating to a wide variety of ES. Here we used InVEST to model changes in carbon sequestration and storage, water yield, nitrogen retention and export, crop production and recreation (S1 Appendix). The water yield and nutrient retention models have recently been tested and validated using contemporary data in the UK [42,43]. Where an InVEST model was unavailable for a particular ES or was considered unsuitable, an extended benefit transfer approach was utilized incorporating indices based on the land cover map categories linked to ES delivery. The following ES were mapped using such proxy values: flood regulation, timber production, livestock production, soil quality, aesthetic value, habitat suitability for pollinators and biodiversity. The proxy values for each land cover type were derived from [24,25] (see S1 Appendix). Using these approaches, ES were mapped for each scenario using the land cover maps as input.

### 2.5. Economic impact

To examine the potential economic impacts of land cover change, scenario development was supported by use of an input-output economic model. This is a conventional form of economic model, which is currently being used to inform strategic planning within the study area. To construct the model, we used a combination of Office for National Statistics data and the Cambridge Econometrics Local Economic Forecasting Model ([44]) for the Dorset area for the period 1981–2015. Model outputs included traditional economic metrics of Gross Value Added (GVA) and full-time equivalent (FTE) employment (see S2 and S3 Appendices). The model was used to estimate changes in GVA and employment for each scenario by assuming that agricultural productivity varied in direct proportion to the total area of agricultural land. All other model inputs remained constant for the different scenarios.

While input-output models are widely used to support economic planning, they do not explicitly consider links with the environment. For this reason we performed an additional set of calculations that factored in variation in the demands of different economic sectors for different ES. For this analysis, we employed values describing the dependence of different economic sectors on ES provision provided by Watson and Newton [45], based on a survey of 212 Dorset businesses drawn from 28 different sectors (S4 Appendix). In this survey, dependencies of businesses on flows of different ES were elicited on a five-point Likert scale, ranging from 0 (“not at all dependent”) to 1 (“entirely dependent”). A dependency value of zero implies that a change in flow of a particular ES would have no effect on business performance. In contrast, a dependency value of one suggests that business performance would vary in direct proportion to changes in flow of that ES. In this way, these dependency values provide a measure of demand for different ES. On this basis, for each scenario we adjusted the inputs of each economic sector to the input-output model, by combining dependency values with changes in ES flows. To achieve this, we first calculated the total flow in each ES by summing values across the entire study area, using the results of the ES assessment. Relative changes in ES flow for each scenario were then calculated as a proportion of the initial values. The values of relative change in each ES were then multiplied by the mean dependency values for each individual economic sector obtained from [45]. These values were then summed to provide a combined measure of change in business performance, taking into account changes in all ES and the dependency of different economic sectors upon them. This set of calculations was conducted using Netlogo [46]. The analysis was performed for each scenario, and values were expressed relative to those obtained from the BAU scenario, to provide percentage change values for each economic sector. These were then entered into the input-output model to examine impacts on GVA and employment.

## 3. Results

### 3.1. Land cover change

In the 1930s, the landscape of Dorset was dominated by semi-natural grassland pastures, with neutral unimproved grassland accounting for ~41% of the total area together with additional swathes of calcareous grassland (19%) and smaller patches of acid grassland (1.7%). Heathland (5.5%), broadleaved woodland (8%) and arable cropland (17.8%) were also significant land cover types [34,35]. By 2015 (the BAU scenario), semi-natural grassland (combining the neutral unimproved, calcareous and acid grassland categories) had declined to approximately 1% of the total area (Table 1). Areas of heathland and broadleaved woodland had also declined by 2015 to values of 1.5% and 3.6% respectively. Conversely, the area of improved grassland increased from zero in the 1930s to 45% by 2015, and arable cropland increased to 30% by the latter date, as a result of agricultural expansion and intensification in the region. Coniferous plantations also increased from 0% to 3.7% over the same period, coincident with this intensification process.

**Fig 1:**
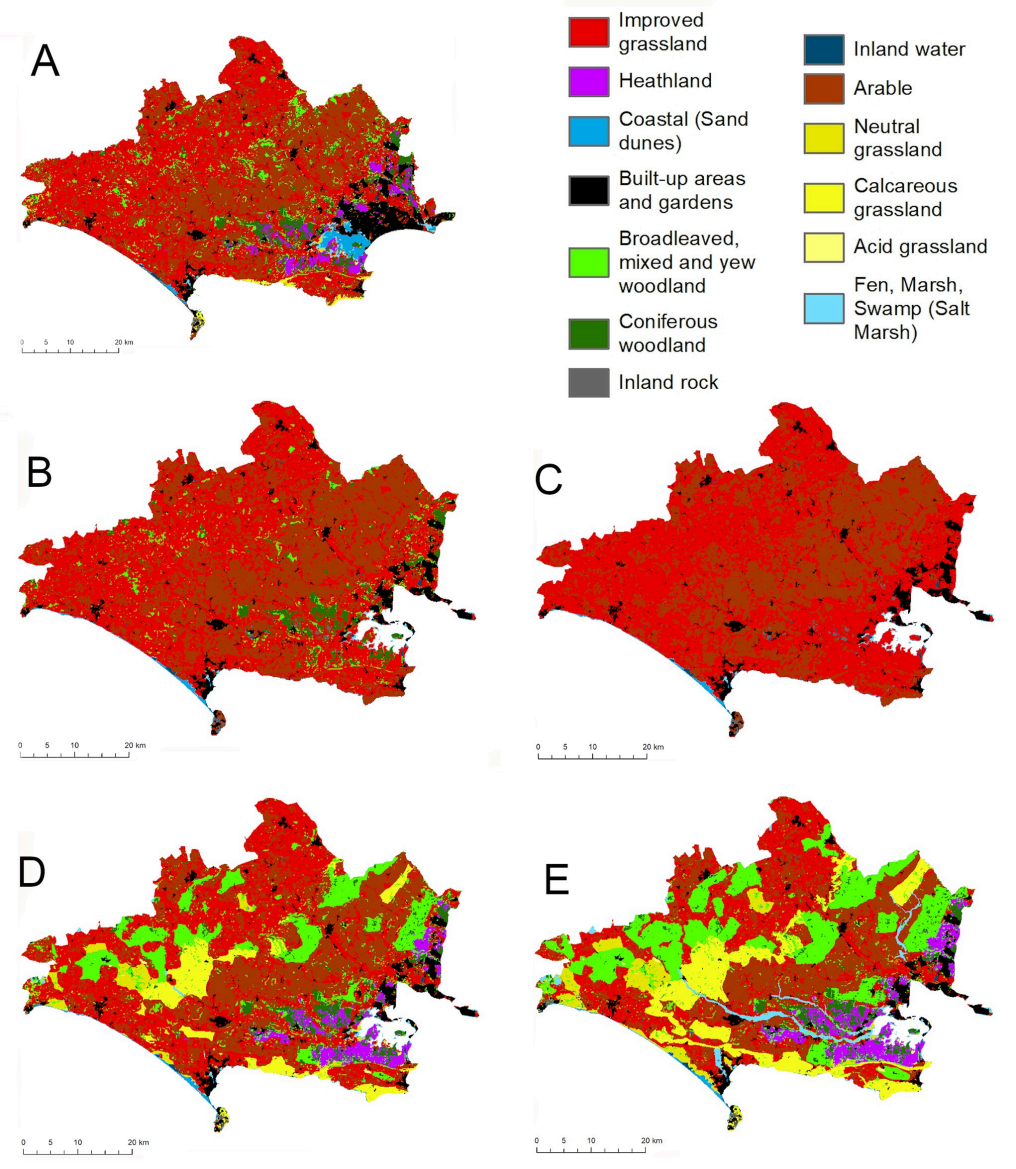
The spatial pattern of land cover change in the different scenarios. (a) BAU: “Business as usual”, (b) (d) (b) LIAB “Agribrexit”, Low Intensity; (c) HIAB “Agribrexit”, High Intensity; (d) LIGB “Green Brexit”, Low Intensity; (e) HIGB “Green Brexit”, High Intensity.

In the HIAB and LIAB scenarios, arable cropland increased only slightly relative to the BAU scenario, with values of 32.3% and 31.7% respectively. Improved grassland increased by slightly larger amounts, giving values of 56.7% and 50.5%. This illustrates the limited scope for further agricultural expansion within the study area; most of the land that is currently of value for agriculture has already been converted (Fig 1). The two scenarios also differed in the amount of broadleaved and coniferous woodland, with combined values of around 6.8% in LIAB and zero in HIAB (Table 1). In contrast, in the HIGB and LIGB scenarios, broadleaved woodland area increased from 6.24% in the BAU to values of 20% and 13.9% respectively, both values exceeding the area present in 1930. Areas of neutral grassland, heathland and calcareous grassland also increased significantly under both scenarios, reaching respective values of 3.64%, 5.18% and 10.4% under HIGB. These increments were associated with corresponding declines in arable cropland and improved grassland (Table 1). The projected habitat changes under HIGB and LIGB varied spatially according to underlying bedrock and soil characteristics; while expansion of broadleaved woodland and calcareous grassland was primarily associated with the chalk landscapes of the western and northern parts of the county, heathland expansion was limited to the acidic gravels and sands of south-eastern areas (Fig 1).

### 3.2. Habitat fragmentation

**Fig 2:**
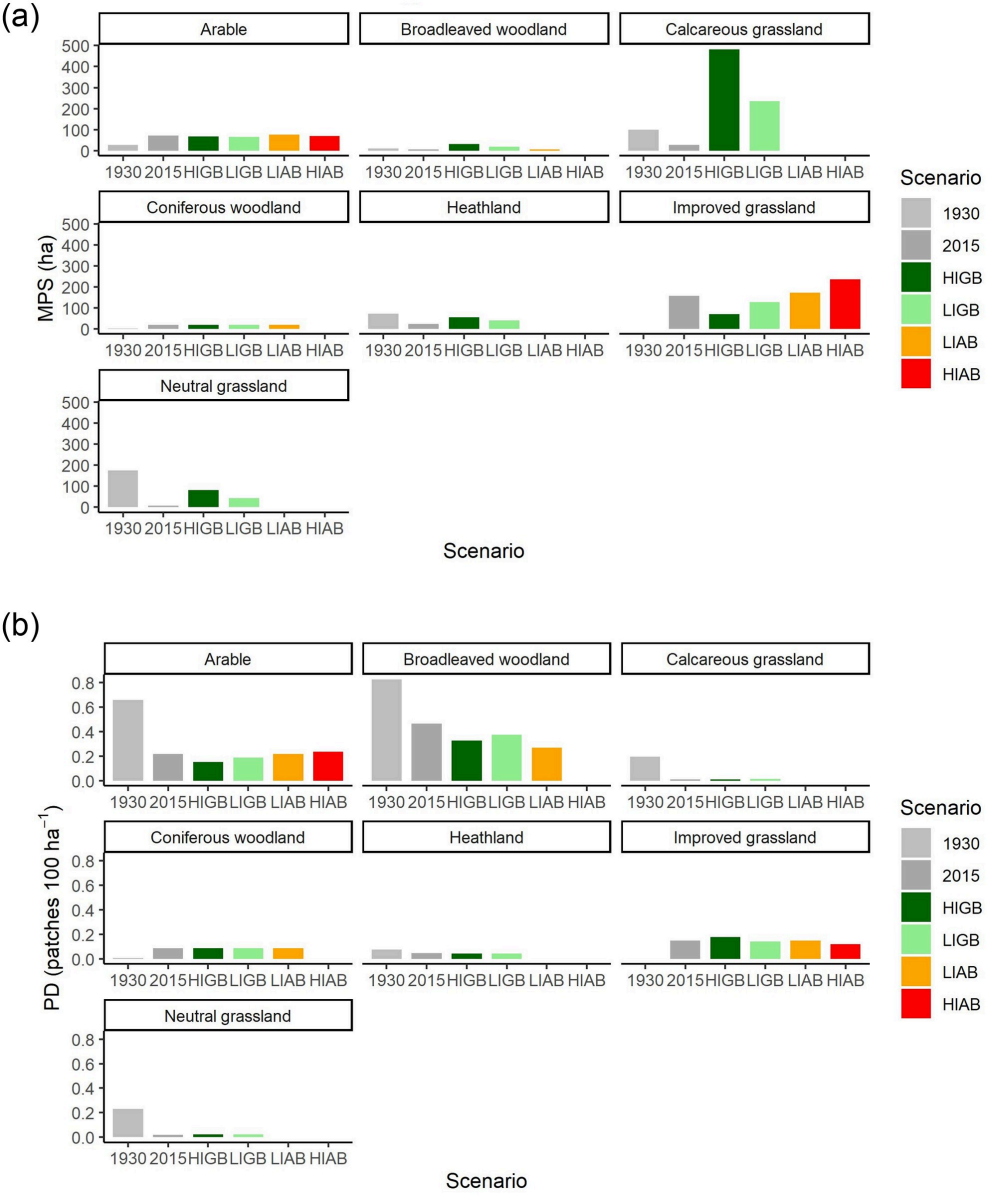
Fragmentation of different land cover types, as determined using FRAGSTATS (see text). (a) Mean Patch Size (MPS), (b) Patch Density (PD).

The expansion and intensification of agriculture in the study area between 1930 and 2015 caused significant fragmentation of semi-natural habitats with high value for biodiversity conservation, including calcareous grassland, broadleaved woodland, heathland and neutral grassland. For example, Mean Patch Size values of these habitats declined by 73%, 32%, 67% and 96% respectively, during this interval. Patch Density also declined in all four semi-natural habitats between 1930 and 2015, by values of 94%, 44%, 39% and 91% respectively (Fig 2). Arable cropland and improved grassland demonstrated corresponding increases in Mean Patch Size and Patch Density over the same timescale, with the exception of Patch Density of arable cropland, which declined by 67%. This likely reflects a trend of increasing size of both arable fields and farms during the process of agricultural intensification.

In the agricultural intensification scenarios, fragmentation continued to intensify, such that Mean Patch Size of the four semi-natural habitats each reached a value of zero under HIAB. Conversely, under the restoration scenarios, Mean Patch Size values increased in all four of these habitats, especially under the HIGB scenario (Fig 2a). These increases were particularly pronounced in calcareous grassland, where values significantly exceeded those from 1930 under both HIGB and LIGB; the same was true for broadleaved woodland. However, in heathland and neutral grassland, Mean Patch Size under either restoration scenario failed to reach the values recorded in 1930. Differences between the scenarios in terms of Patch Density were somewhat less pronounced. For example, although Patch Density values for calcareous grassland were higher in the restoration scenarios than those for agricultural expansion, they were still much lower than those recorded for 1930 (Fig 2b). This likely reflects the focus of the restoration plans used here on restoring large contiguous blocks of land, with the aim of establishing functional habitat networks, rather than increasing the number of discrete habitat patches. Patch Density values for the other semi-natural habitats (i.e. broadleaved woodland, heathland and neutral grassland) showed a similar pattern. Similar results were observed with the other metrics of fragmentation (Table 2); the restoration scenarios tended to increase values of edge density, shape index and core area for habitats of high conservation value, the increments being particularly noticeable for the latter metric. Restoration also increased connectivity between habitats in some cases, such as heathland and calcareous grassland, but this was less evident for broadleaved woodland as indicated by a slight decline in values of the nearest-neighbour metric (Table 2).

**Table 2:** Assessment of habitat fragmentation using FRAGSTATS (see text). (a) For 1930 (b) For the future scenarios. BAU is business as usual, LI/HI = low/high intensity, and AB/GB = Agri-Brexit/Green-Brexit.

Land cover type	ED	MSI	MCA	NN	IJI	
Acid grassland	2.10	1.38	3.64	418	67.7	
Arable cropland	15.7	1.31	11.6	341	54.7	
Broadleaved woodland	10.9	1.22	3.01	367	62.5	
Calcareous grassland	12.9	1.67	49.7	272	51.8
Coastal	0.18	1.67	2.68	2334	51.1	
Coniferous woodland	0.04	1.05	0	5053	66.9
Fen, marsh, swamp	0.15	1.38	5.85	1062	69.9	
Heathland	2.78	1.44	42.6	318	69.4	
Inland water	1.42	1.12	0.45	510	62.2	
Neutral grassland	18.9	1.53	101	267	66.5	
Land cover type	Scenario	ED	MSI	MCA	NN	IJI
Acid grassland	BAU	0.12	1.18	1.94	1620	55.2
Arable cropland	BAU	9.42	1.39	42.9	354	28.8
Broadleaved woodland	BAU	5.34	1.20	1.08	3756	47.9
Calcareous grassland	BAU	0.24	1.41	9.26	922	68.8
Coniferous woodland	BAU	1.65	1.29	7.70	601	62.8
Fen, marsh, swamp	BAU	0.15	1.21	0.64	481	76.2
Heathland	BAU	0.98	1.29	10.8	504	67.2
Improved grassland	BAU	11.9	1.41	93.8	284	45.1
Neutral grassland	BAU	0.22	1.21	0.97	1090	64.0
Arable cropland	HIAB	9.90	1.34	42.9	341	9.59
Fen, marsh, swamp	HIAB	0.21	1.21	0.64	481	64.2
Improved grassland	HIAB	10.6	1.33	164	287	24.3
Arable cropland	LIAB	9.49	1.37	46.4	343	13.3
Broadleaved woodland	LIAB	2.65	1.18	0.73	437	32.3
Coniferous woodland	LIAB	1.65	1.29	7.70	601	53
Fen, marsh, swamp	LIAB	0.15	1.21	0.64	481	59.3
Improved grassland	LIAB	12.6	1.35	104	285	46.4
Acid grassland	HIGB	0.12	1.18	1.94	1620	77
Arable cropland	HIGB	5.92	1.37	41.2	401	36.7
Broadleaved woodland	HIGB	5.10	1.20	22.1	386	78.2
Calcareous grassland	HIGB	1.61	1.71	370	473	63.6
Coniferous woodland	HIGB	1.65	1.29	7.70	601	56.1
Fen, marsh, swamp	HIGB	0.72	1.39	15.8	528	77.3
Heathland	HIGB	1.59	1.38	28.7	436	66.7
Improved grassland	HIGB	7.16	1.42	39.4	297	50.3
Neutral grassland	HIGB	0.88	1.35	53.7	901	74.8
Acid grassland	LIGB	0.12	1.18	1.94	1620	72.2
Arable cropland	LIGB	7.59	1.38	39.6	353	31
Broadleaved woodland	LIGB	5.27	1.20	10.6	385	66.2
Calcareous grassland	LIGB	0.93	1.56	180	671	65.6
Coniferous woodland	LIGB	1.65	1.29	7.70	601	61.6
Fen, marsh, swamp	LIGB	0.22	1.24	1.46	676	83.7
Heathland	LIGB	1.26	1.34	20.7	481	67.0
Improved grassland	LIGB	9.47	1.47	75.6	287	47.0
Neutral grassland	LIGB	0.64	1.30	25.1	856	70.6

Abbreviations: ED, Edge Density; MSI, Mean Shape Index; MCA, Mean Core Area; NN, Euclidean Nearest-Neighbour distance; IJI, Interspersion and Juxtaposition Index.

### 3.3 Ecosystem services

**Fig 3:**
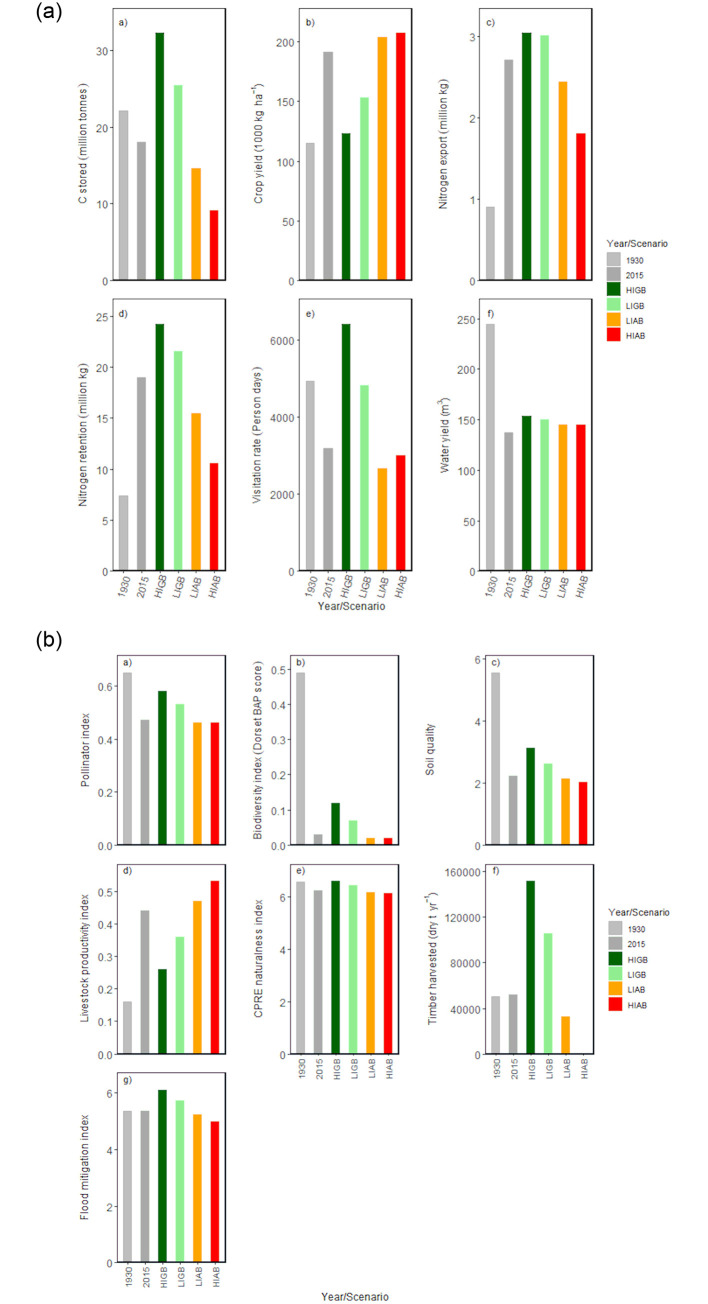
Ecosystem service (ES) provision summarized at the landscape scale, for each of the different scenarios, and including values for the 1930s as a historic reference. Values for 2015 are equivalent to the BAU scenario. (**3a**) (a) carbon sequestration and storage, (b) arable crop production, (c) nutrient (nitrogen) export, (d) nutrient retention, (e) recreation value (assessed as visitation rate), (f) water yield, (**3b**) (a) habitat quality for pollinators, (b) biodiversity value, using index for BAP species, (c) soil quality, (d) livestock production, (cows, poultry, sheep, pigs), (e) aesthetic value (CPRE index), (f) timber production, (g) flood mitigation.

Variation in the provision of ES between the different scenarios differed markedly depending on the ES concerned (Fig 3). As expected, crop yield and livestock production were higher under HIAB and LIAB than under the restoration scenarios. However, for every other ES, provision was higher under HIGB and LIGB than under the agricultural expansion scenarios; values under the restoration scenarios were also consistently higher than the BAU scenario (equivalent to 2015). In other words, ecological restoration increased ES flows compared to the current situation in every case except for those ES associated with agricultural production. Furthermore, with the exception of crop and livestock production, ES flows were consistently higher under HIGB than under LIGB. Some ES flows under HIGB and LIGB also exceeded values recorded for 1930; this was the case for carbon storage, nitrogen export and retention, timber production, and flood mitigation. However, the value of the biodiversity index was far higher in 1930 than under any of the other scenarios; the highest value of the pollinator index was also observed for 1930.

### 3.4 Economic impact

**Fig 4:**
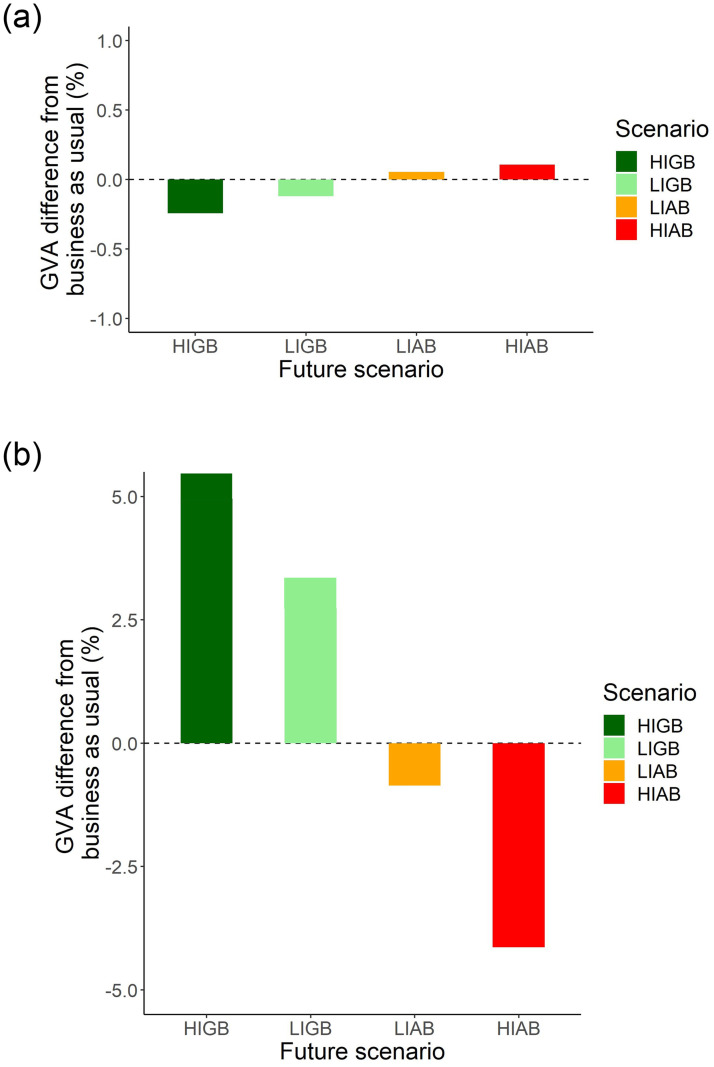
Economic impacts (GVA) associated with the different scenarios, relative to the BAU value. (a) using the unadjusted input—output model, (b) using the input-output model adjusted for ecosystem service flows.

Using the unadjusted input-output model, the scenarios had only a slight economic impact in terms of the forecast change in GVA. While the ecological restoration scenarios had a slight negative impact on GVA (≤ -0.25%), the agricultural expansion scenarios resulted in a slight increase (≤ 0.25%). In each case, the impacts were larger for the higher intensity scenario than for the lower intensity (Fig 4a). Conversely, when the input-output model was adjusted to take account of ES flows, GVA under the LIGB and HIGB increased by 3.0% and 5.4% respectively, whereas under LIAB and HIAB GVA was reduced by 0.9% and 4.5% respectively.

The current employment in Dorset, according to the Dorset Local Economy Forecasting Model for Dorset County Council 2016/7, is approximately 380,000. The largest sectors are retail (17%), health and social work (15.3%), manufacturing (9%) and accommodation and food services (9%). Agriculture and forestry currently account for approximately 0.2% of full time employment. The scenarios developed here using the unadjusted input-output model differed very little in terms of employment; LIAB and HIAB registered slight increases of 0.12% and 0.24% respectively, while LIGB and HIGB were associated with slight decreases of -0.28% and -0.57% respectively. Conversely, when the input-output model was adjusted to take account of ES flows, employment under the LIGB and HIGB increased by 3.7% and 6.7% respectively, whereas under LIAB and HIAB employment was reduced by 1.1% and 5.6% respectively.

Overall, for both GVA and employment, these results indicate that the ranking of the scenarios was reversed when ES flows were factored in. The magnitude of forecast economic impacts was also substantially higher when ES flows were included in the analysis (Fig 4b).

## 4. Discussion

This research delivered two key insights. First, the contribution of rural land use to the economy is much larger than that provided by agriculture alone; patterns of land use affect the performance of multiple economic sectors by influencing ES flows to a wide variety of different businesses. This influence of agricultural land use on the wider economy is ignored by conventional approaches to economic forecasting, which take no account of ES flows and their importance to business performance. Second, landscape-scale ecological restoration of agricultural land can potentially increase the contribution of this land to economic development and employment, by increasing flows of all ES other than those associated with agricultural production. The evidence provided by this research therefore supports calls for investment in natural capital, using approaches such as ecological restoration and rewilding, as a way of strengthening economic performance while also providing benefits to human well-being [47–50].

The fact that economic planning typically ignores the value of natural capital, and the associated flows of ES, has long been recognised. Indeed, the original development of ES as a concept was stimulated by this problem. In response, recent global initiatives such as the Millennium Ecosystem Assessment [51], The Economics of Ecosystems and Biodiversity [52] and the International Science-Policy Platform on Biodiversity and Ecosystem Services (IPBES) [53] have developed conceptual frameworks to support the valuation of nature and to better capture these values in decision-making. As a result of such initiatives, the concepts of natural capital and ecosystem services are now broadly accepted, as is their potential contribution to improving environmental management [27]. However, despite growing awareness of these concepts, their practical application is still limited, and the core problem has not been fully addressed. Economic objectives continue to be of overriding importance for land-use decision-making, regardless of the type of decision-maker involved [54].

The widespread failure of land-use decision makers to recognise the value of natural capital may partly reflect the type of evidence that is available. Although an immense body of research has been conducted into natural capital and ES over the past 20 years, most of this has focused only on the supply side of the economy, and not on demand [28]. Consequently, there is still very little understanding of how land-use decisions taken at the local scale influence economic performance at regional or national scales. In their review of recent progress in ES research, Costanza et al. [27] highlight the need for integration of information across scales, and note the importance of developing full cost accounting in business and governmental sectors, which would enable the positive contributions of ES to businesses and households to be identified. Yet progress in this area has been very limited to date. As a consequence, little evidence regarding natural capital and ES is available in a form that can directly influence land-use decision making. This is illustrated by the information needs of stakeholders in the study area that we examined here, namely Dorset. The strategic economic plan for this region focuses primarily on only two goals: strengthening economic growth (as indicated by GVA) and employment; no consideration is given to the need to invest in natural capital or to maintain ES flows [55]. This is despite the fact that 8–10% of Dorset’s economy, and some 30,000 jobs, are directly dependent on the state of the local environment; employment in agriculture, forestry and fisheries accounts for about a quarter of this total [56]. Information on trends in ES values has little traction with such decision-makers; rather, an explicit linkage needs to be made between the state of natural capital and both economic development and employment.

As we demonstrate here, models describing the dynamics of natural capital and ES flows can potentially be linked with conventional economic models through the use of shared parameters. These can then be used to support the development of scenarios of future land use, which can inform decision-making. This approach represents a form of loose coupling between models, where the output from one model is used as input to another. Such an approach has previously been identified as a useful way of developing modelling ‘toolkits’, enabling information to be integrated across a range of scales, and used to support strategic landscape planning [57,58]. Clearly, this form of loose coupling could potentially be achieved with a wide variety of different modelling approaches. For example, a number of alternative methods for mapping ES are available other than those employed here, including MIMES, LUCI and ARIES [40]. Similarly, a range of different approaches are available for modelling the economy, including Computable General Equilibrium (CGE), Dynamic Stochastic General Equilibrium, and system dynamics models [59]. Although an input-output economic model was employed here, to be consistent with current economic planning in the study area, this approach does not fully capture the complexity of the linkages between human society and the natural environment. As noted by Costanza et al. [27], more integrated, dynamic, non-linear systems models are needed to achieve this, but their use in supporting land use planning has been very limited to date.

Few researchers have examined how ES models might best be linked with economic models. Recent reviews of ES modelling and mapping approaches (e.g. [60–62]) fail to give any consideration to the issue. Conversely, Drechsler [63] reviews recent progress in developing mechanistic models that integrate ecological and socio-economic knowledge, including agent-based models, bio-economic models, ecological-economic models, social-ecological models and system-dynamic models, but does not consider how these might best be linked with spatially explicit ES models. The most detailed examination is provided by Anger et al. [59], who note that while some studies have assessed the macroeconomic performance of certain environment-related sectors (especially agriculture, forestry and fisheries), they have generally not considered the impact of changes in multiple ES on measures of macroeconomic performance, such as GVA and employment. Examples of relevant studies include the preliminary CGE analysis presented by Bosello et al. [64] on the macroeconomic effects of changes of selected ES provided by European forest, cropland and grassland ecosystems, but focusing primarily on carbon sequestration. Further, Ukidwe and Bakshi [65] explored the contribution of ecosystems to energy flows in 91 sectors of the US economy, but did not include impacts on macroeconomic indicators. Building on the National Ecosystem Assessment undertaken in the UK, Bateman et al. [66] used a combination of process-based and econometric regression models to estimate the value of ES provision under different land use scenarios at the national scale. However, no attempt was made to evaluate the demand for ES flows from different sectors of the economy, or the impact on economic growth. These examples illustrate the scope for linking ES and economic models, but also highlight the need for further research in this area, building on the approach outlined here.

As noted by Rieb et al. [60], another key knowledge gap relates to identifying the beneficiaries of ES. Few ES models identify the beneficiaries of a given ES in a spatially explicit way, or map the connections between supply and demand. Understanding how ES supply, which is spatially explicit, relates to demand, which is often spatially disaggregated, has been identified as a research priority in order to better meet stakeholder needs [67,68]. In the study area examined here, results indicated that the economy is geographically structured: most businesses are located in urban areas, which are concentrated in the south-east part of the county. Yet most ES are produced in rural areas. Economic performance of the county is therefore at least partly dependent on ES flows from rural to urban areas, a process that has been little examined. This highlights the need to understand the urban-rural interface in relation to natural capital and ES flows, to ensure that approaches to regional development planning are sustainable. In particular, there is a need to ensure that urban development does not negatively affect the supply of rural ES, and that the natural capital of rural areas is adequately protected to ensure sustainable provision of ES to urban areas [69,70].

In common with all other exploratory scenario-based analyses, those presented here are based on a number of assumptions. The most important of these is the assumption that business performance is linearly related to ES flows, as captured by the ES dependency values obtained from a questionnaire survey. The potential limitations of the questionnaire data are explored by Watson and Newton [45]. Chief among these is the issue of sampling; it is conceivable that a different set of dependency values would have been obtained from a different set of respondents. Although the response rate to the survey was evenly distributed across the different economic sectors, and stratified sampling approaches were adopted, the sample may have been biased in different ways. This limitation could potentially be addressed by more comprehensive business surveys, including more detailed assessment of how business performance is linked to ES flow [45]. Analysis of variation in dependency values would enable sensitivity analyses to be performed, allowing the impact of this variation on economic forecasts to be explored. A further assumption is that the ES flows used by businesses were derived only from the study area; although many businesses were found to place a high importance on using locally produced resources [45], it is possible that ES flows from other areas might have influenced business performance. Although issues such as inter-regional ES flows and telecoupling [71] were not examined here, they could influence the extent to which business performance is linked with local natural capital. Furthermore, the flows of different ES were combined additively, and weighted by the dependency values; in reality, business performance might display non-linear relationships with ES flows, and conceivably different ES flows might interact. For example, problems with low water quality are likely to be more intense when water flows are low, owing to drought [72]. In addition, no consideration was given here to potential changes over time in the condition of natural capital, which could be caused by factors such as climate change. Finally, it should be noted that the case study area selected here, namely Dorset, may not be representative of all lowland agricultural landscapes in the region; this might limit the generality of the conclusions drawn. Given these limitations, the analyses presented here should clearly be viewed as preliminary, and the results viewed with caution.

Despite these caveats, these results suggest that ecological restoration or rewilding of agricultural land could benefit the economy through increased provision of ES flows, with GVA increases of up to around 5% projected in the scenarios explored. We did not differentiate here between ecological restoration and rewilding as approaches to expand the area of semi-natural habitats; potentially, either could be implemented. Large-scale rewilding is often considered to be more applicable to relatively marginal areas, where the economic value of land is low, such as uplands or mountainous areas [12,14,18]. However, initiatives such as the Knepp Estate in the UK demonstrate that rewilding is also an economically viable land use option in lowland agricultural landscapes [73]. Analyses presented by Loth and Newton [74] demonstrate strong interest among stakeholders for rewilding in Dorset, with naturalistic grazing and farmland abandonment emerging as the most suitable rewilding options following spatial multi-criteria analysis. However, it is important to note that rewilding could lead to very different outcomes than other ecological restoration approaches, even if their broad goals are similar [75]. For example, rewilding might result in woodland expansion on successional habitats, such as heathland and calcareous grassland, which are currently deemed to be of high biodiversity value. It should also be recognised that increases in flows of some ES from agricultural land can be achieved through means other than restoration or rewilding, for example by improved husbandry of crops and livestock, as advocated in approaches such as “regenerative agriculture” and “sustainable intensification” [76,77].

Whichever approach is implemented, the large-scale expansion of semi-natural habitats is consistent with the vision described in a number of policy initiatives in the UK, including the development of a resilient ecological network [78] and the UK Government’s 25 Year Environment Plan, which aims to restore 500,000 ha of wildlife-rich habitat throughout the territory, and to achieve a 12% increase in woodland cover in England by 2060 [79]. In particular, the current results support suggestions that following Brexit, the UK should develop new land use policies that enable landowners to receive government funds for the provision of ES [29,80]. Specifically, in a review of post-Brexit land use policy options, Helm [80] has identified the payment of public monies for the provision of public goods as the preferred approach, as it would enable the production of a wide range of public benefits. Our results indicate that the provision of financial incentives to landowners to implement ecological restoration or rewilding approaches could not only provide benefits to wildlife and people, but could strengthen the contribution of rural land to economic development and employment. As noted by Helm [80], if this policy option is pursued, it will need to be supported by a comprehensive evaluation of land use and its impact on the provision of benefits to the public. Potentially the approaches outlined here could contribute to achievement of this goal.

## Supporting information

S1 Appendix InVEST and ecosystem service modelling methodology. (DOCX)

S2 Appendix An input-output model of the Dorset economy—Methodology. (DOCX)

S3 Appendix Dorset input-output model—Spreadsheet. (XLSM)

S4 Appendix Ecosystem service dependency values—Spreadsheet. (XLSX)
