# Supplementary material for: Ecological restoration of agricultural land can improve its contribution to economic development
Source: PLoS One. 2021 Mar 5;16(3):e0247850. doi: 10.1371/journal.pone.0247850 (PMC7935295; doi:10.1371/journal.pone.0247850)
Supplement: S1 Appendix — (DOCX) [file pone.0247850.s001.docx]

**Appendix 1**

**InVEST and Ecosystem Service Modelling Methodology**

1. **Carbon**

The InVEST (v3.4.4) Carbon Storage and Sequestration: Climate Regulation model was used to map carbon storage within each broad habitat (Figure S1). Carbon pools data for input into the model were extracted from Jiang *et al.* (2013) and the median taken for each landcover type for above ground stored carbon in biomass (Mg ha^-1^), below ground stored carbon in biomass (Mg ha^-1^), carbon stored in soil and carbon stored in dead organic matter in Mg ha^-1^ (Table S1). The sum of these categories estimates the carbon stock *per* 1-ha grid cell. Jiang *et al.* (2013) used different broad habitat categories to our maps (1930, 1950 and 2015) so to fill in the InVEST carbon table we combined, averaged or excluded some of the classifications as follows:

- Bogs, fens, marsh, swamp and saltmarsh were averaged and combined to form “Fen, Marsh, Swamp (Salt Marsh)”
- Supra-littoral and Supra Littoral sediment (= littoral sediment) became Coastal (Sand dunes)
- Urban and sub urban became “Built-up areas and gardens”
- Montane habitats (= peatland) was not available on any of the new maps, so was excluded.

**Table S1: Pooled carbon pool data extracted from Jiang *et al.* (2013) for the county of Dorset. Values are median values or each habitat type. Unidentified habitats and waters were given a value of zero.**

| LCM broad habitats | Carbon pools (Mg ha^-1^) | | | |
| --- | --- | --- | --- | --- |
|  | Above ground | Below ground | Soil | Dead materials |
| Acid grassland | 6 | 6 | 85.87 | 2 |
| Arable | 3.01 | 2 | 50.7 | 0 |
| Broadleaved, mixed and yew woodland | 116 | 120 | 111.5 | 55 |
| Built-up areas and gardens | 0 | 0 | 25 | 0 |
| Calcareous grassland | 3 | 6 | 20 | 2 |
| Coastal (Sand dunes) | 5.61 | 0 | 0 | 0 |
| Coniferous woodland | 94.75 | 110 | 95.5 | 50 |
| Fen, Marsh, Swamp (Salt Marsh) | 7.54 | 3.55 | 49.51 | 0.5 |
| Heathland | 7 | 5.5 | 64.99 | 1.5 |
| Improved grassland | 3 | 2.5 | 21.5 | 0.5 |
| Inland rock | 5.61 | 0 | 0 | 0 |
| Inland water | 2.82 | 0 | 76.2 | 0 |
| Neutral grassland | 3 | 4 | 70.3 | 1 |

Net carbon change was estimated as the difference per 1-ha grid cell in total carbon stock between each period.

1. **Annual Water Yield**

The InVEST (v3.4.4) Annual Water Yield model was used to represent the relative contributions of water from different parts of the landscape of Dorset, offering insight into how changes in land use patterns have affected annual surface water yield. Gridded estimates (1 km) of monthly areal rainfall (mm) for Dorset (1930-2015) were obtained from the UK CEH-GEAR dataset (Tanguy *et al.,* 2015) and monthly estimates converted to mean annual values.

The reference potential evaporation estimates for the model were evaluated against the Global Potential EvapoTranspiration dataset (Global-PET: Trabucco and Zomer, 2009) provided by the Consultative Group for International Agricultural Research (CGIAR) which is derived from the WorldClim dataset (Hijmans *et al.,* 2005). We used the annual climatology that is representative for the period 1950-2000 available at a resolution of 30 arc seconds. Unfortunately, the WorldClim data set is representative for a period further forward in time for the 1930’s period and further back for the 2015 period. We assume that between 1930-1950 and 2000-2015 the reference evapotranspiration and the crop coefficient for each grid square were similar. These estimates were however, refined in the final version of the model by calculating the seasonality constant (Z) which captures the local precipitation pattern and additional hydrogeological characteristics of the catchments during the 1930 and 2015 periods. Z was calculated using the formula n*0.20 (as per Redhead *et al.,* 2016), where n is the average number of rain days ( > 1 mm) over the study period (i.e. between 1930-2015). N was estimated at approximately 170 for the southwest of the UK based on Radcliffe Meteorological Station data ([www.geog.ox.ac.uk/research/climate/rms/rainday.html](http://www.geog.ox.ac.uk/research/climate/rms/rainday.html)), giving a value of 33.8 for Z.

A depth to root restricting layer was downloaded from the European Soil Database (ESDB), as derived data for ‘Depth available to roots’ in cm (Panagos *et al.,* 2012; Hiederer 2013a, 2013b). This was then reclassified using the ‘Reclassify’ spatial analyst tool to convert the cm measurements into mm, as the data needs of the model dictated. Plant available water content (PAWC) was also needed as a raster as fraction values between 0 and 1. The fraction was obtained through a division of the available water content (in mm) divided by soil depth (Sharp *et al.,* 2015). Available water content was averaged from top soil and sub soil available water content (mm) 1 km x 1 km rasters from European Soil Database Derived data (Panagos *et al.,* 2012; Hiederer 2013a, 2013b). The Raster calculator was used to calculate mean available water content across the top soil and sub soil rasters. This was divided by the root restricting layer to give a Plant Available Water Content (PAWC) fraction raster with all values between 0 and 1.

Watershed data was obtained from the Environment Agency (2015) from the Water Framework Directive River Waterbody Catchments Cycle 2 as polygon dataset, which includes 12 coastal catchments for Dorset allowing the full extent of the case study area to be modelled. Catchments are defined as an area of land from which all surface run-off flows through a series of streams, rivers and, possibly, lakes to a particular point in the water course such as a river confluence. As the InVEST water yield model calculates at the watershed level, the watersheds that fully or partially intersected the study area boundary were selected. The InVEST model also requires several tabular values for each broad habitat class. These include whether the land cover class is vegetated or not, rooting depth and a plant evapotranspiration coefficient (Kc). We estimated these coefficients for each of the board habitats by matching class descriptions in Redhead *et al.* (2016).

**3. Nutrient retention and export**

To calculate the retention and export of nitrogen in terrestrial vegetation across the Dorset area the InVEST (v.3.4.4) the nutrient delivery ratio (NDR) model was used. The main outputs from the NDR model reflect the per-pixel load of Total Nitrogen (TN), modified by the ability of vegetation to retain nutrients (with units of kg year^-1^), accounting for both surface and subsurface retention. The second main output is nutrient export (with units of kg year^-1^), representing mean annual nutrient delivery to streams, and maps showing per-pixel and watershed contribution to nutrient yield. In the study area examined here, nitrogen deposition has increased in recent decades, owing to the application of fertilizers to farmland. This has led to increases in both the amount of nitrogen retained by vegetation, and the amount exported to water bodies.

The NDR model had the same data needs as the Water Yield model, with the addition of a biophysical table, with water quality coefficients including nitrogen loading, and vegetation filtering values for each pixel. Nutrient retention coefficients for each broad habitat class were obtained from Redhead *et al.* (2018) who previously performed an extensive literature search for values relevant to the UK broad habitats classes. Historic nitrogen loading values (N ha^-1^ yr^-1^) for each broad habitat class in Dorset were obtained from CEH modelled data and the nitrogen atmospheric Concentration Based Estimated Deposition (CBED) data (Smith *et al.,* 2018; Tipping *et al.,* 2017). Additionally, the Nutrient Retention model required the Flow Accumulation rate from InVEST (v.3.5.0) RouteDEM. We used the Ordnance Survey 50 m Digital Terrain Model (DTM) (Ordnance Survey (GB) 2015) for the DEM. The ‘Fill’ spatial analyst tool in ArcMap (v10.1 © ESRI UK Limited, Aylesbury) was then used to eliminate sinks and combined with a digital watercourse network to ensure routing along known watercourses. The InVEST RouteDEM v 3.5.0 (Natural Capital Project 2015) tool was then run with the updated DEM with multiple levels of threshold flow accumulation between 10 and 2500, to calculate a threshold value for flow accumulation (TFA). A visual appraisal found the 1350 threshold flow accumulation level being the most similar to the OS Mastermap in terms of waterways (OS Open Data 2016).

**4. Flood regulation**

The capacity of vegetation to mitigate flood risk was assessed utilising a scoring approach using a non-monetary valuing technique developed by Hodder *et al.* (2010). Differences in land cover will affect flood risk through effects on surface roughness or infiltration capacity, which will affect water retention rates, and hence the volume and timing of flow (Nelson *et al.,* 2009). Following Hodder *et al.* (2010) land cover classes were each given a score based on expert judgement (Table S2). The index used by Hodder *et al.* (2010) is based on multiple factors in the form of:

$$\boldsymbol{Land Use Flood Risk=}\frac{\boldsymbol{Altitude*}\boldsymbol{Slope*Standard Perc}\boldsymbol{entage Runoff}}{\boldsymbol{Moisture Retention Index}}$$

This method assumes that flood risk would be a direct consequence of land cover and makes the assumption that an increase in the moisture retention index value at a given point will decrease the risk of excess storm driven runoff. We make one amendment to the index, by changing the value of the “Arable” Retention Index Value from 8 to 3. This is based on more recent evidence that transformation of permanent grassland to arable land reduces pore rigidity and mechanical soil compaction generally implying lower condition of the soil and the ability to retain moisture (Ajayi *et al.,* 2016).

**Table S2. Soil Moisture Retention Index Values from Hodder *et al.,* (2010). High values represent high potential to maintain a low soil moisture deficit. *Realigned in this study.**

| LCM broad habitats | Water retention index |
| --- | --- |
| Acid grassland | 8 |
| Arable | 3* |
| Broadleaved, mixed and yew woodland | 10 |
| Built-up areas and gardens | 6 |
| Calcareous grassland | 4 |
| Coastal (Sand dunes) | 8 |
| Coniferous woodland | 9 |
| Fen, Marsh, Swamp (Salt Marsh) | 10 |
| Heathland | 4 |
| Improved grassland | 6 |
| Inland rock | 2 |
| Neutral grassland | 6 |
| Inland water | 10 |

**5.Timber production**

It is possible to estimate a theoretical, sustainable maximum yield that could be produced from the management of Dorset’s woodlands using a set of co‐efficients produced by Forest Research (Forestry Commission, 2008). This sustainable wood yield value is that which can be harvested which is available for conventional markets.

The equation given to calculate the approximate biomass yield as a function of yield class is:

***Biomass yield = a + ( b * yield class)***

The parameters for these equations for Dorset have been calculated by Munro *et al.* (2009) as shown below:

**Table S3 Parameters of equations relating biomass to yield class**

| Woodland type | a | b | Avg. Yield class in England | Sustainable yield (t/ha/yr) |
| --- | --- | --- | --- | --- |
| Broadleaved | 1.151 | 0.2874 | 5 | 2.588 |
| Conifers | 0.095 | 0.1255 | 12 | 1.601 |

This analysis assumed that trees in Dorset achieve the average yield class for England. Based on these assumptions the maximum sustainable yield from Dorset woodlands in this study is detailed in Table S4.

**Table S4 Maximum sustainable yield from Dorset woodlands**

| Woodland type | Cover (ha) | % of total | Sustainable yield (t/ha/yr) | Total yield (t/yr) |
| --- | --- | --- | --- | --- |
| Broadleaved (1930) | 19423.16 | 99.78 | 2.59 | 50305.984 |
| Broadleaved (1950) | 18517.14 | 99.77 | 2.59 | 47959.393 |
| Broadleaved (1980) | 15076.5 | 64.85 | 2.59 | 39048.135 |
| Broadleaved (2015) | 14857.45 | 63.9 | 2.59 | 38480.796 |
| Conifers  (1930) | 41.2 | 0.21 | 1.6 | 65.92 |
| Conifers  (1950) | 41.85 | 0.22 | 1.6 | 66.96 |
| Conifers  (1980) | 7437.39 | 31.99 | 1.6 | 11899.824 |
| Conifers  (2015) | 8390.07 | 36.09 | 1.6 | 13424.112 |

**6. Soil Quality**

Owing to lack of historic directly measured soil quality data at the regional scale, estimated soil erosion rates were used to reclassify land cover using a proxy method based on work by Graves *et al.* (2015) who previously estimated soil erosion rates in England and Wales by land use/soil type category. Erosion rates for 4 soilscapes (clay, silt, sand and peat) recorded by Graves *et al.* (2015) were then averaged and unity-based normalized using the following formula, Z = normalised value, and X = existing value: to create a “soil quality” index with values ranging between 0-1.

$$\boldsymbol{Z=}\frac{\boldsymbol{X-}\boldsymbol{min(X)}}{\max\left( \boldsymbol{X} \right)\boldsymbol{-min(X)}}$$

The approach used is similar to that used by the eSOTER project (<http://www.esoter.org>) in which erosion rates represent an average for specific land use practices. As agricultural practices were notably less intensive in the 1930s relative to the rest of the periods, two index values were used to represent arable soil erosion potential. The “Arable extensive” (meaning: to obtaining a relatively small crop from a large area with a minimum of capital and labour) rate was used for the 1930s, while the “Arable intensive” (Intensive farming practices include growing high-yield crops, using fertilisers and pesticides and keeping animals indoors) was used for the 1950s onwards. These values were then aligned to each Dorset land use category in ArcGIS.

**Table S5 Estimated erosion rates (t ha^−1^ a^−1^) in Dorset by land use** **category**

| LCM broad habitats | Normalised erosion rates (t ha^−1^ a^−1^) |
| --- | --- |
| Arable extensive | **0.31** |
| Arable intensive | **1.00** |
| Coastal Sand dunes and inland rock | **0** |
| Grassland improved | **0.23** |
| Grassland unimproved | **0.21** |
| Horticulture | **0.64** |
| Inland Water | **0** |
| Neutral grassland | **0.15** |
| Urban | **0.22** |
| Wildscape (includes heathland) | **0.03** |
| Woodland | **0.03** |

**7. Recreation**

The InVEST (v3.4.4) Visitation: Recreation and Tourism model was used to map potential recreational activity across the county. The purpose of the InVEST recreation model is to predict the spread of person-days of recreation and tourism, based on the locations of natural habitats. In the absence of empirical data on visitation, we parameterized the model using a proxy for visitation: geotagged photographs posted to the website flickr (Yahoo, 2018) were used to calculate average photo user days for the period 2015 period for each 1-ha grid cell (Natural Capital Project, 2015) within the Dorset county boundary. As this method was only viable for the 2015 period, these estimates were combined with the Alternative Scenario Analysis component of the model which allowed us to predict how historic changes to the landscape will alter visitation rate using a least squares regression. Scenario predictors represented the same broad habitat features that were selected as Predictor Variables, but represented modified versions of those features based on the area of each broad habitat present in each time period. The model does not presuppose that any predictor variable (e.g. the changes in the broad habitat) has an effect on visitation. Instead, the tool estimates the magnitude of each predictor’s effect based on its spatial correspondence with current visitation in the area of interest. In developing these accounts, it was found that the urban habitat classification “Built-up areas and gardens” excluded most green spaces (e.g. public gardens, parks, golf courses) and therefore would not provide information about the extent of green space in the urban environment and the interaction between society and the environment in these areas that would be of most use to policy. Due to this we did not include this category in our analysis.

**8. Aesthetic value** **‘naturalness’**

Aesthetic value was assessed using scores based on aesthetic attributes identified from the CPRE Tranquility Mapping study (Jackson *et al.,* 2008). As the perception of aesthetic qualities is very subjective, it is therefore important to select indicators based on robust testing using a large sample size with wide coverage. This study was selected because it was based on a substantial survey of UK public (4000 people) and the indicators used were spatially linked to aesthetic features. The method has an underlying assumption that perceived ‘naturalness’ is an aesthetic benefit and accepts that naturalness may be perceived rather than actual/ecological naturalness (Tveit *et al.,* 2006). In practice, the CPRE ‘naturalness’ scores, with a range of 0-10 where 10 is extremely natural, were aligned to LCM habitat types (Table S6). The overall score of naturalness for each period was calculated as:

$$\boldsymbol{Naturalness}\mathbf{=}\frac{\boldsymbol{Area*}\boldsymbol{Natur}\boldsymbol{alness score}}{\boldsymbol{Total Area}}$$

**Table S6 Aesthetic/naturalness index for broad habitat classifications developed by interpretation of the land classes used in the CPRE Tranquility Mapping study (Jackson *et al,* 2008).**

| LCM broad habitats | Naturalness score |
| --- | --- |
| Acid grassland | 7 |
| Arable | 5 |
| Broadleaved, mixed and yew woodland | 7.5 |
| Built-up areas and gardens | 3.3 |
| Calcareous grassland | 7 |
| Coastal (Sand dunes) | 9 |
| Coniferous woodland | 7 |
| Fen, Marsh, Swamp (Salt Marsh) | 9 |
| Heathland | 8 |
| Improved grassland | 7 |
| Inland rock | 9 |
| Neutral grassland | 7 |
| Inland water | 9 |

**9. Habitat quality for pollinators**

Spatial values for nectar productivity, species nectar diversity and functional nectar diversity by UK broad habitat classification were extracted from Baude *et al.* (2016). Baude *et al.* (2016) used a combination of two national scale vegetation surveys of Britain, so the study is applicable to the county of Dorset. Baude *et al.* (2016) found close parallels between historical changes in pollinator communities and changes in nectar resources. This suggests that using the nectar productivity and species and functional nectar diversity data is appropriate as a proxy for pollinator communities. Baude *et al.* (2016) do not state that one of these measures is more important as an indicator for pollinators; hence all three measures were normalised and weighted equally to create a combined index of habitat quality for pollinators (Table S7). Despite not including urban habitats, Baude *et al.* (2016) acknowledge its contribution to national nectar provision. Urban gardens are important as contributors of richness and composition of flora, and cover 18-27% of UK urban areas (Loram *et al.* 2008). To include urban areas within this study, pollinator abundance was scaled from farmland values from a study by Baldock *et al.* (2015). Their study used 36 field sites in 12 locations across the UK, with urban land located within cities, and the matched farmland within 10 km of the city sampled (Baldock *et al.,* 2015).

**Table S7 Habitat quality index for pollinators in Dorset**

|  |  | Original values | | | | Normalised values | | | Habitat Quality Index |
| --- | --- | --- | --- | --- | --- | --- | --- | --- | --- |
| Broad Habitats | **Match from Baude *et al.,* (2016)** | **Nectar Productivity** | | **Species nectar diversity** | **Functional diversity** | **Nectar Productivity** | **Species nectar diversity** | **Functional diversity** | **Average values** |
| Acid grassland | Acid grassland | 29.38 | 0.78 | | 0.44 | 0.301 | 0.50 | 0.70 | 0.50 |
| Arable | Arable | 6.9 | 0.61 | | 0.3 | 0.07 | 0.4 | 0.47 | 0.31 |
| Broadleaved, mixed and yew woodland | Broadleaf | 70.04 | 1.03 | | 0.63 | 0.72 | 0.67 | 1 | 0.8 |
| Built-up areas and gardens | Data from Badlock *et al.,* (2015) | 4.17 | 0 | | 0 | 0.04 | 0 | 0 | 0.01 |
| Calcareous grassland | Calcareous grassland | 97.48 | 1.54 | | 0.54 | 1 | 1 | 0.86 | 0.95 |
| Coastal (Sand dunes) | - | - | 0 | | 0 | 0 | 0 | 0 | 0 |
| Coniferous woodland | Conifer | 14.49 | 0.71 | | 0.41 | 0.15 | 0.45 | 0.65 | 0.42 |
| Fen, Marsh, Swamp (Salt Marsh) | Fen,Bog, Saltmarsh | 39.22 | 0.95 | | 0.54 | 0.40 | 0.62 | 0.86 | 0.63 |
| Heathland | Shrub Heath | 82.43 | 0.72 | | 0.39 | 0.85 | 0.47 | 0.62 | 0.64 |
| Improved grassland | Improved grassland | 51.73 | 0.73 | | 0.48 | 0.53 | 0.47 | 0.76 | 0.6 |
| Inland rock | - | - | 0 | | 0 | 0 | 0 | 0 | 0 |
| Neutral grassland | Neutral grassland | 64.84 | 1.03 | | 0.58 | 0.67 | 0.67 | 0.92 | 0.76 |
| Inland water | Freshwater | 0 | 0 | | 0 | 0 | 0 | 0 | 0 |

**10. Biodiversity**

This analysis focused on species of conservation concern, namely those listed on the UK Biodiversity Action Plan (BAP). Nationally the UK BAP, identifies habitats and species that are of principal importance in the UK. To establish the likely importance of BAP species, occurring in different broad habitats of Dorset, a species richness indicator developed by Newton *et al.* (2012) was used to reclassify land cover (Table S8). Values of both BAP species number and density were based on records made within the Frome catchment area of Dorset and subsequently normalised (score from 0 to 1) to provide measures of local biodiversity. It is notable that BAP species priorities and policies are likely to have changed over the last 85 years. Nonetheless such proxies provide a contemporary based estimate of how the impact of habitat change has impacted the refuges for biodiversity across Dorset.

**Table S8** Species richness index from Newton *et al.* (2012). The total number of BAP species recorded for each land cover type is presented, together with species density values, calculated by dividing the total number of species by the area of each land cover type. These values were then normalised on a scale of 0-1.

| Land cover type | Total number of BAP species recorded | Species density (number of BAP species per hectare) | Dorset BAP species richness index | Dorset Priority BAP Habitat |
| --- | --- | --- | --- | --- |
| Acid grassland | 40 | 0.087 | 0.369 | ✓ |
| Arable | 77 | 0.01 | 0.014 | ✓(Arable land) |
| Broadleaved, mixed and yew woodland | 114 | 0.03 | 0.105 | ✓ |
| Built-up areas and gardens | 51 | 0.033 | 0.12 |  |
| Calcareous grassland | 62 | 0.051 | 0.202 | ✓ |
| Coastal (Sand dunes) | 1 | 0.062 | 0.253 | ✓ |
| Coniferous woodland | 52 | 0.03 | 0.106 |  |
| Fen, Marsh, Swamp | 11 | 0.107 | 0.459 | ✓ |
| Heathland | 92 | 0.044 | 0.172 | ✓ |
| Improved grassland | 110 | 0.007 | 0 | ✓ |
| Inland rock | 22 | 0.037 | 0.139 |  |
| Neutral grassland | 66 | 0.224 | 1 | ✓ |
| Inland water | 3 | 0.08 | 0.335 | ✓ |

**References**

Ajayi, A.E. and Horn, R., (2016). Transformation of ex-arable land to permanent grassland promotes pore rigidity and mechanical soil resilience. Ecological Engineering, 94, 592-598.

Baldock, K. C., Goddard, M. A., Hicks, D. M., Kunin, W. E., Mitschunas, N., Osgathorpe, L. M., Potts, S. G., Robertson, K. M., Scott, A. V. and Stone, G. N., (2015). Where is the UK's pollinator biodiversity? The importance of urban areas for flower-visiting insects. Proceedings of the Royal Society of London B: Biological Sciences, 282, (1803), 1-10.

Baude, M., Kunin, W.E., Boatman, N.D., Conyers, S., Davies, N., Gillespie, M.A., Morton, R.D., Smart, S.M. and Memmott, J., (2016). Historical nectar assessment reveals the fall and rise of floral resources in Britain. Nature, 530(7588), 85.

Bullock, J. M., Jefferson, R.G., Blackstock, T.H., Pakeman, R.J., Emmett, B.A., Pywell, R.J., Grime, J.P., Silvertown, J., (2011). Semi-natural Grasslands. In: The UK National Ecosystem Assessment Technical Report. Cambridge: UNEP-WCMC.

Crofts, A. and Jefferson, R. G., (1999). Lowland Grassland Management Handbook. Sheffield, UK: English Nature/The Wildlife Trusts.

Defra (2015) The June Agricultural Survey data (1980-2015) Available from: [www.defra.gov.uk/esg/work_htm/publications/cs/farmstats_web](http://www.defra.gov.uk/esg/work_htm/publications/cs/farmstats_web) [Accessed 02/08/2018].

Environment Agency, (2015). WFD River Waterbody Catchments Cycle 2 [online]. Available from: https://data.gov.uk/dataset/wfd-river-waterbody-catchments-cycle-2 [Accessed 04/09/2018].

Forestry Commission, (2008). Introduction and background In: Management Plan for the Crown Lands of the New Forest (Part B). Lyndhurst, UK: Forestry Commission.

Graves, A.R., Morris, J., Deeks, L.K., Rickson, R.J., Kibblewhite, M.G., Harris, J.A., Farewell, T.S. and Truckle, I., (2015). The total costs of soil degradation in England and Wales. Ecological Economics, 119, 399-413.

Hiederer, R., (2013a). Mapping soil properties for Europe: spatial representation of soil database attributes. Luxembourg: Publications Office of the European Union.

Hiederer, R., (2013b). Mapping Soil Typologies: Spatial Decision Support Applied to the European Soil Database. Luxembourg: Publications Office of the European Union.

Hijmans, R.J., Cameron, S.E., Parra, J.L., Jones, P.G. and Jarvis, A., (2005). Very highresolution interpolated climate surfaces for global land areas. International journal of climatology, 25(15), 1965-1978.

Hodder, KH., Douglas S., Newton, A., Bullock, JM., Scholefield, P., Vaughan, R., Cantarello, E., Birch, J., (2010) Building and evaluating alternative management scenarios. Appendix 1 to Final report to Defra. Defra Competition Code: WC0758/CR0444. Bournemouth University. Available: [www.randd.defra.gov.uk/Document.aspx?Document=WC0758_10012_FRA.pdf](http://www.randd.defra.gov.uk/Document.aspx?Document=WC0758_10012_FRA.pdf) Accessed: [26/07/2018]

Jackson, S., Fuller, D., Dunsford, H., Mowbray, R., Hext, S., MacFarlane, R. and Haggett, C., (2008). Tranquillity Mapping: developing a robust methodology for planning support. Report to the Campaign to Protect Rural England, Centre for Environmental and Spatial Analysis, Northumbria University, Bluespace environments and the University of Newcastle upon on Tyne.

Janssens, F., Peeters, A., Tallowin, J., Bakker, J., Bekker, R., Fillat, F. and Oomes, M., (1998). Relationship between soil chemical factors and grassland diversity. Plant and soil, 202 (1), 69-78.

Jiang, M., Bullock, J.M. and Hooftman, D.A., (2013). Mapping ecosystem service and biodiversity changes over 70 years in a rural English county. Journal of Applied Ecology, 50(4), 841-850

Loram, A., Warren, P.H. and Gaston, K.J., (2008). Urban domestic gardens (XIV): the characteristics of gardens in five cities. Environmental Management, 42(3), 36

Munro, T (2009) Technical study –   Woodfuel supply and demand in Dorset . For Dorset Woodlink. Available[**https://www.cse.org.uk/downloads/file/Dorset%20woodlink%20study%20final%20version%202.pdf**](https://www.cse.org.uk/downloads/file/Dorset%20woodlink%20study%20final%20version%202.pdf) Accessed [21/11/2018]

Nelson, E., Mendoza, G., Regetz, J., Polasky, S., Tallis, H., Cameron, D., Chan, K.M., Daily, G.C., Goldstein, J., Kareiva, P.M. and Lonsdorf, E., (2009). Modeling multiple ecosystem services, biodiversity conservation, commodity production, and tradeoffs at landscape scales. Frontiers in Ecology and the Environment, 7(1),4-11.

Newton, A.C., Hodder, K., Cantarello, E., Perrella, L., Birch, J.C., Robins, J., Douglas, S., Moody, C. and Cordingley, J., (2012). Cost–benefit analysis of ecological networks assessed through spatial analysis of ecosystem services. Journal of Applied Ecology, 49(3), 571-580.

Nix, J., (2018). John Nix farm management. John Nix farm management., (Ed. 48).

OS Open Data (2015). OS Terrain 50 Map (1:50 000) [online]. EDINA Digimap Ordnance Survey Service. Available from: <https://www.ordnancesurvey.co.uk/business-and-government/products/terrain-50.html>

OS Open Data, (2016). OS Strategic Map (1: 100,000) [online]. EDINA Digimap Ordnance Survey Service. Available from: <http://digimap.edina.ac.uk/>

Panagos, P., Van Liedekerke, M., Jones, A. and Montanarella, L., (2012). European Soil Data Centre: Response to European policy support and public data requirements. Land Use Policy, 29 (2), 329-338.

Redhead, J.W., May, L., Oliver, T.H., Hamel, P., Sharp, R. and Bullock, J.M., (2018). National scale evaluation of the InVEST nutrient retention model in the United Kingdom. Science of the Total Environment, 610, 666-677.

Sharp, R., Tallis, H. T., Ricketts, T., Guerry, A. D., Wood, S. A., Chaplin-Kramer, R., Nelson, E., Ennaanay, D., Wolny, S., Olwero, N., Vigerstol, K., Pennington, D., Mendoza, G., Aukema, J., Foster, J., Forrest, J., Cameron, D., Arkema, K., Lonsdorf, E., Kennedy, C., Verutes, G., Kim, C. K., Guannel, G., Papenfus, M., Toft, J., Marsik, M., Bernhardt, J., Griffin, R., Glowinski, K., Chaumont, N., Perelman, A., Lacayo, M., Mandle, L., Hamel, P., Vogl, A. L., Rogers, L. and Bierbower, W., (2015). InVEST 3.2.0 User’s Guide. Stanford, CA, USA: The Natural Capital Project, Stanford University, University of Minnesota, The Nature Conservancy, and World Wildlife Fund.

Smith, R.I.; Dore, A.J.; Tang, Y.S.; Stedman, J.R. (2018). Sulphur and nitrogen atmospheric Concentration Based Estimated Deposition (CBED) data for Dorset (1930-2015). NERC Environmental

Tanguy, M.; Dixon, H.; Prosdocimi, I.; Morris, D. G.; Keller, V. D. J. (2015). Gridded estimates of daily and monthly areal rainfall for the United Kingdom (1890-2014) [CEH-GEAR]. NERC Environmental Information Data Centre. <https://doi.org/10.5285/f2856ee8-da6e-4b67-bedb-590520c77b3c>

Tavener, L.E., (1952). Changes in the Agricultural Geography of Dorset, 1929-49. Transactions and Papers (Institute of British Geographers), (18),93-106.

The Natural Capital Project., (2015). InVEST 3.4.4 documentation - Visitation: Recreation and Tourism [online]. The Natural Capital Project: Available from: <http://data.naturalcapitalproject.org/nightly-build/invest-users-guide/html/recreation.html#rec-how-it-works>[Accessed 02/08/2018].

Tipping, E., Davies, J.A.C., Henrys, P.A., Kirk, G.J.D., Lilly, A., Dragosits, U., Carnell, E.J., Dore, A.J., Sutton, M.A., Tomlinson, S.J., (2017). Long-term increases in soil carbon due to ecosystem fertilization by atmospheric nitrogen deposition demonstrated by regional-scale modelling and observations. Scientific reports, 7(1), 1-11.

Trabucco, A. and Zomer, R.J., (2009). Global aridity index (global-aridity) and global potential evapo-transpiration (global-PET) geospatial database. CGIAR Consortium for Spatial Information.

Tveit, M., Ode, Å. and Fry, G., (2006). Key concepts in a framework for analysing visual landscape character. Landscape research, 31(3),229-255.

Wood, S.A., Guerry, A.D., Silver, J.M. and Lacayo, M., (2013). Using social media to quantify nature-based tourism and recreation. Scientific reports, 3, 2976.

Yahoo, (2018). Flickr, a yahoo company [online]. Available from: <https://www.flickr.com>[Accessed 02/08/2018].
