# Supplementary material for: Ecological restoration of agricultural land can improve its contribution to economic development
Source: PLoS One. 2021 Mar 5;16(3):e0247850. doi: 10.1371/journal.pone.0247850 (PMC7935295; doi:10.1371/journal.pone.0247850)
Supplement: S2 Appendix — (DOCX) [file pone.0247850.s002.docx]

# Appendix 2. An Input-Output Model of the Dorset Economy – Methodology

## Input Output Model

The basis for the Dorset Input Output model is the 2013 Analytical Input-output table (OAT) for the UK. The first step in ‘regionalising’ this table is to estimate a ‘Domestic Use’ table – in essence, one that shows industry purchases from the UK market in its intermediate section. The second step is to estimate the local purchase elements of this table (and thereby regional imports and exports).

A report containing a description of the theory and assumptions behind the 2010-2015 OATs and their practical implementation is available in the ONS achieve depository (Wild 2010). The ‘Extended Input-Output Model of the Dorset Economy’, was designed by the South West Economy Centre of the University of Plymouth. The model is based on a set of economic and social accounts that allow analysis of the structure of and relationships within the Dorset economy. Economic impact analysis (EIA) using this model takes into account not only the direct impact of a change on a particular industry, but also the ripple effects in the economy. These are brought about by changes in local spending patterns by the industry affected and also alterations to household incomes and consumption expenditure. The sum of these changes results in a net effect across the whole Dorset economy that can be expressed in terms of change in demand for output, GVA or FTE. The model also provides a sectoral analysis allowing identification of those sectors that are most affected by a particular change or shock to the economy. The basis for input into the Dorset Input Output model was the 2015 period GVA and FTE data representing the industry sectors as having relatively strong links to the environment (which are highlighted in yellow on the model spreadsheet). The intermediate and sections of this table are scaled according to the GVA market share of Dorset industries representing the purchases of goods and services from the UK market scaled by Dorset’s share of UK GVA.

In common with other Input-Output models, the model depicts inter-industry relationships within the economy, which incorporates linkages between sectors. Specifically, output from one industrial sector may become an input to other industrial sectors. These relationships between sectors are captured in a series of equations embedded within the model, which represent the relationships between the output of a first industry and the inputs it must receive from other industries to produce its own output. Employment calculations are based on productivity values, which are calculated empirically for each separate industrial sector as the ratio of the size of the labour force to the output value. Projections of changes in employment (Table 3) are calculated by multiplying these ratios with the changed output values determined by the model. Therefore employment values vary among scenarios as a function of the changing economic performance of different sectors and their interrelationships as represented in the model. However, was it is assumed that the productivity values remain constant for each sector, for each scenario.

FTE Employment

- Full time and part time employees are taken from the Business Register (and its predecessors) for Dorset and GB.
- Agricultural employment (and self employment) are derived from DEFRA data. The latest available data for Dorset is 2009 – from thereon changes are assumed to follow the path of the NUTS1 South West region as detailed in the Business Register.
- Data for military employees are taken from DASA records.
- Data for persons serving domestic households (SIC group 97) are taken from the Annual Population Survey (APS) for GB and the NUTS1 SW region. Dorset’s share of employment is estimated according to its share of SW GVA in SIC group 97.
- Self employment is estimated at 4 SIC digit level for GB from the APS. The intensity of self employment in GB industries is applied at the local level. This is scaled to the estimate of total self-employment for Dorset given by the APS.
- The conversion from headcount to FTE is achieved by applying the hours worked by 4-digit industry estimated at a GB level from the APS. Hours worked are estimated for full and part time employees and the self-employed. The basis for one FTE is 37 hours.
- Using these data, the ratio of FTE/OUTPUT was calculated for each sector, and these values were used to forecast the impact of economic changes on employment.

Gross Value Added

- GB data on GVA for the Input-Output groups is derived from the UK National Accounts, Supply and Use Tables. The GB share of UK employment is used to derive a first estimate of GVA by I-O group SIC and this is then constrained to the GB estimates of GVA that appear in ONS Regional Accounts.
- ONS Regional Accounts GVA data is taken from the Dorset and Somerset NUTS2 region, covering 32 industry groups. Productivity in each of the 32 industry groups in Dorset and Somerset is then estimated (following the employment enumeration methodology detailed above).
- A first estimate of GVA by I-O group for Dorset is derived by applying GB productivities to the I-O level FTE employment data for Dorset. Estimates of Dorset GVA in 32 industries are then derived by applying the productivities implied by the Dorset and Somerset NUTS2 data. These are used to constrain the I-O group estimates derived from GB productivities. Finally, these estimates are constrained to the ONS Regional Accounts GVA in Dorset for 11 industries.

### The ‘Domestic Use’ Table

- The intermediate and sections of this table are scaled according to the GVA market share of Dorset industries, i.e. each column of the table representing the purchases of goods and services from the UK market by industry *j* is scaled by Dorset’s share of UK GVA in the *j*th industry.
- The UK household demand vector is scaled according to Dorset’s share of UK Gross Domestic Household Income (GDHI), taken from ONS Regional Accounts. Leakages through income tax, social contributions *etc* are not represented in the primary input section of the UK I-O tables. Estimates of these elements for Dorset are therefore taken from Dorset’s GDHI data and added to the primary input section of the regional model.
- Government expenditures in Dorset are derived by applying Dorset’s ‘market share’ of UK expenditure, i.e. expenditure by government in each industry *i* is estimated as Dorset’s share of UK GVA in the *i*th industry.
- Total Gross Domestic Fixed Capital Formation (GDFCF) in Dorset is taken from ONS Regional Accounts estimates at the NUTS2 level and applying Dorset’s share of the NUTS2 area’s GVA. The vector of GDFCF in the UK I-O table is scaled to this total, thus forming a vector of GDFCF for Dorset.
- Service exports (non-tourist) are estimated using the same market share assumption used to derive Government expenditures. These estimates are adjusted to reflect the available NUTS1 data on service exports (largely resulting in a downward scaling of the market share estimates).
- The total expenditures of overnight-staying tourists within the Dorset region is derived from the relevant Domestic and International tourism surveys. The pattern of their expenditures is derived from UK Supply and Use tables and the Input-Output tables for Scotland.
- International Exports are derived according to the market share principle, using Dorset’s share of UK GVA in each *i* industry.
- Compensation of employment (CoE) and mixed income by industry are estimated as follows. Compensation of employment by industry in Dorset is initially estimated from UK CoE by applying Dorset’s share of UK GVA in each industry. These estimates are then constrained to ONS Regional Accounts estimates of CoE in Dorset for 11 industries. An initial estimate of mixed income by Dorset industry is formed by applying CoE per employee to the number of self-employed in each industry. These estimates are subsequently constrained to ONS Regional Accounts estimates of mixed income in Dorset across 11 industries. Together the estimates of CoE and mixed income form the row of Primary Inputs that links to the Household expenditure column of final demand.

### Estimation of Local Purchases and Regional Trade

- At this stage of estimation, a ‘residual’ column can be derived from the gross output of industry *i* minus its implied intermediate and final demand sales. This column represents net regional trade in each *i* industry and as such its elements can be negative (i.e. where the implied intermediate and final demand sales exceed the gross output of the *i* th industry). In order to form a balanced regional I-O table however each of the *i* elements of the residual vector must be non-negative. This is achieved by lowering the elements of the *i* th row, effectively implying that some of the purchases of *i*’s output are imported from the rest of the UK. The resulting residual vector can then be interpreted as a vector of regional exports.
- ONS Regional Accounts do not record estimates of regional trade. Typically therefore estimates need to be derived artificially. There are many ‘standard’ mechanical techniques for doing this (e.g. the Simple Location Quotient method). However, in instances where regional trade data have been estimated by survey (e.g. the Scottish I-O tables), such methods have been shown to be highly inaccurate.
- The regional trade propensities for Dorset are therefore established rather more subjectively, the aim being to arrive at ‘credible’ trading propensities. Firstly, the survey-based regional import and export propensities for Scotland are considered in relation to the Scottish industry’s market share of UK output. The Dorset trade propensities are set in relation to this evidence, the relative specialisation of industries in Dorset, and the general view that as a smaller economy, Dorset will tend to be more open to trade than the Scottish economy. Given that households and firms spend through different market channels (e.g. households tend to purchase through retailers rather than through direct suppliers), separate local consumption/regional import propensities are allowed for each.
- Table 2 shows the local import propensities implied by the Simple Location Quotient (SLQ) method and those within the Dorset I-O model. Generally the table shows that the SLQ would assume a much higher local consumption propensity than those derived by the method above. Indeed wherever the Location Quotient is above 1 (i.e. the Dorset sector is relatively specialised) the SLQ method assumes Dorset firms and households buy everything locally (100% local consumption propensity). This of course is unrealistic and is certainly not reflected in Scottish trade data. The model’s propensities are considered to be a more credible reflection of local consumption propensity than the SLQ.

**Reference**

Wild, R (2010), United Kingdom Input-Output Analytical Tables 2010. Office for National Statistics report. Available: https://webarchive.nationalarchives.gov.uk/20160114044923/http://www.ons.gov.uk/ons/rel/input-output/input-output-analytical-tables/2010/index.html (Accessed 02/07/2020)

## Table 1 – UK Input Output Classifications by SIC

| **SIC** | **INDUSTRY** |
| --- | --- |
| 01 | Products of agriculture, hunting and related services |
| 02 | Products of forestry, logging and related services |
| 03 | Fish and other fishing products; aquaculture products; support services to fishing |
| 05 | Coal and lignite |
| 06&07 | Extraction Of Crude Petroleum And Natural Gas & Mining Of Metal Ores |
| 08 | Other mining and quarrying products |
| 09 | Mining support services |
| 10.1 | Preserved meat and meat products |
| 10.2-3 | Processed and preserved fish, crustaceans, molluscs, fruit and vegetables |
| 10.4 | Vegetable and animal oils and fats |
| 10.5 | Dairy products |
| 10.6 | Grain mill products, starches and starch products |
| 10.7 | Bakery and farinaceous products |
| 10.8 | Other food products |
| 10.9 | Prepared animal feeds |
| 11.01-6 and 12 | Alcoholic beverages & Tobacco products |
| 11.07 | Soft drinks |
| 13 | Textiles |
| 14 | Wearing apparel |
| 15 | Leather and related products |
| 16 | Wood and of products of wood and cork, except furniture; articles of straw and plaiting materials |
| 17 | Paper and paper products |
| 18 | Printing and recording services |
| 19 | Coke and refined petroleum products |
| 20A | Industrial gases, inorganics and fertilisers (all inorganic chemicals) - 20.11/13/15 |
| 20B | Petrochemicals - 20.14/16/17/60 |
| 20C | Dyestuffs, agro-chemicals - 20.12/20 |
| 20.3 | Paints, varnishes and similar coatings, printing ink and mastics |
| 20.4 | Soap and detergents, cleaning and polishing preparations, perfumes and toilet preparations |
| 20.5 | Other chemical products |
| 21 | Basic pharmaceutical products and pharmaceutical preparations |
| 22 | Rubber and plastic products |
| 23.5-6 | Cement, lime, plaster and articles of concrete, cement and plaster |
| 23OTHER | Glass, refractory, clay, other porcelain and ceramic, stone and abrasive products - 23.1-4/7-9 |
| 24.1-3 | Basic iron and steel |
| 24.4-5 | Other basic metals and casting |
| 25.4 | Weapons and ammunition |
| 25OTH | Fabricated metal products, excl. machinery and equipment and weapons & ammunition - 25.1-3/25.5-9 |
| 26 | Computer, electronic and optical products |
| 27 | Electrical equipment |
| 28 | Machinery and equipment n.e.c. |
| 29 | Motor vehicles, trailers and semi-trailers |
| 30.1 | Ships and boats |
| 30.3 | Air and spacecraft and related machinery |
| 30OTH | Other transport equipment - 30.2/4/9 |
| 31 | Furniture |
| 32 | Other manufactured goods |
| 33.15 | Repair and maintenance of ships and boats |
| 33.16 | Repair and maintenance of aircraft and spacecraft |
| 33OTH | Rest of repair; Installation - 33.11-14/17/19/20 |
| 35.1 | Electricity, transmission and distribution |
| 35.2-3 | Gas; distribution of gaseous fuels through mains; steam and air conditioning supply |
| 36 | Natural water; water treatment and supply services |
| 37 | Sewerage services; sewage sludge |
| 38 | Waste collection, treatment and disposal services; materials recovery services |
| 39 | Remediation services and other waste management services |
| 41-43 | Construction |
| 45 | Wholesale and retail trade and repair services of motor vehicles and motorcycles |
| 46 | Wholesale trade services, except of motor vehicles and motorcycles |
| 47 | Retail trade services, except of motor vehicles and motorcycles |
| 49.1-2 | Rail transport services |
| 49.3-5 | Land transport services and transport services via pipelines, excluding rail transport |
| 50 | Water transport services |
| 51 | Air transport services |
| 52 | Warehousing and support services for transportation |
| 53 | Postal and courier services |
| 55 | Accommodation services |
| 56 | Food and beverage serving services |
| 58 | Publishing services |
| 59-60 | Motion Picture, Video & TV Programme Production, Sound Recording & Music Publishing services & Programming And Broadcasting services |
| 61 | Telecommunications services |
| 62 | Computer programming, consultancy and related services |
| 63 | Information services |
| 64 | Financial services, except insurance and pension funding |
| 65 | Insurance and reinsurance, except compulsory social security & Pension funding |
| 66 | Services auxiliary to financial services and insurance services |
| 68.1-2 | Real estate services, excluding on a fee or contract basis and imputed rent |
| 68.2IMP | Owner-Occupiers' Housing Services |
| 68.3 | Real estate services on a fee or contract basis |
| 69.1 | Legal services |
| 69.2 | Accounting, bookkeeping and auditing services; tax consulting services |
| 70 | Services of head offices; management consulting services |
| 71 | Architectural and engineering services; technical testing and analysis services |
| 72 | Scientific research and development services |
| 73 | Advertising and market research services |
| 74 | Other professional, scientific and technical services |
| 75 | Veterinary services |
| 77 | Rental and leasing services |
| 78 | Employment services |
| 79 | Travel agency, tour operator and other reservation services and related services |
| 80 | Security and investigation services |
| 81 | Services to buildings and landscape |
| 82 | Office administrative, office support and other business support services |
| 84 | Public administration and defence services; compulsory social security services |
| 85 | Education services |
| 86 | Human health services |
| 87-88 | Residential Care & Social Work services |
| 90 | Creative, arts and entertainment services |
| 91 | Libraries, archives, museums and other cultural services |
| 92 | Gambling and betting services |
| 93 | Sports services and amusement and recreation services |
| 94 | Services furnished by membership organisations |
| 95 | Repair services of computers and personal and household goods |
| 96 | Other personal services |
| 97 | Services of households as employers of domestic personnel |

## Table 2 – Implied Simple Location Quotient Local Purchasing Propensities v Dorset Model

| **SIC** | **INDUSTRY** | **SLQ** | **FIRMS** | **HHOLDS** |
| --- | --- | --- | --- | --- |
| 01 | Products of agriculture, hunting and related services | 100% | 80% | 10% |
| 02 | Products of forestry, logging and related services | 100% | 70% | 10% |
| 03 | Fish and other fishing products; aquaculture products; support services to fishing | 100% | 80% | 10% |
| 05 | Coal and lignite | 0% | 0% | 0% |
| 06&07 | Extraction Of Crude Petroleum And Natural Gas  & Mining Of Metal Ores | 10% | 2% | 0% |
| 08 | Other mining and quarrying products | 100% | 10% | 10% |
| 09 | Mining support services | 100% | 10% | 0% |
| 10.1 | Preserved meat and meat products | 40% | 5% | 5% |
| 10.2-3 | Processed and preserved fish, crustaceans, molluscs, fruit and vegetables | 14% | 10% | 5% |
| 10.4 | Vegetable and animal oils and fats | 0% | 0% | 0% |
| 10.5 | Dairy products | 37% | 10% | 5% |
| 10.6 | Grain mill products, starches and starch products | 100% | 20% | 15% |
| 10.7 | Bakery and farinaceous products | 61% | 10% | 10% |
| 10.8 | Other food products | 100% | 20% | 15% |
| 10.9 | Prepared animal feeds | 64% | 20% | 5% |
| 11.01-6 and 12 | Alcoholic beverages & Tobacco products | 98% | 20% | 15% |
| 11.07 | Soft drinks | 0% | 0% | 0% |
| 13 | Textiles | 96% | 30% | 5% |
| 14 | Wearing apparel | 12% | 5% | 1% |
| 15 | Leather and related products | 48% | 10% | 5% |
| 16 | Wood and of products of wood and cork, except furniture; articles of straw and plaiting materials | 100% | 40% | 20% |
| 17 | Paper and paper products | 43% | 10% | 5% |
| 18 | Printing and recording services | 97% | 50% | 70% |
| 19 | Coke and refined petroleum products | 4% | 0% | 0% |
| 20A | Industrial gases, inorganics and fertilisers (all inorganic chemicals) - 20.11/13/15 | 12% | 5% | 5% |
| 20B | Petrochemicals - 20.14/16/17/60 | 4% | 1% | 0% |
| 20C | Dyestuffs, agro-chemicals - 20.12/20 | 0% | 0% | 0% |
| 20.3 | Paints, varnishes and similar coatings, printing ink and mastics | 100% | 20% | 10% |
| 20.4 | Soap and detergents, cleaning and polishing preparations, perfumes and toilet preparations | 100% | 10% | 10% |
| 20.5 | Other chemical products | 14% | 5% | 5% |
| 21 | Basic pharmaceutical products and pharmaceutical preparations | 5% | 5% | 1% |
| 22 | Rubber and plastic products | 92% | 20% | 10% |
| 23.5-6 | Cement, lime, plaster and articles of concrete, cement and plaster | 48% | 20% | 20% |
| 23OTHER | Glass, refractory, clay, other porcelain and ceramic, stone and abrasive products - 23.1-4/7-9 | 43% | 20% | 20% |
| 24.1-3 | Basic iron and steel | 39% | 15% | 5% |
| 24.4-5 | Other basic metals and casting | 54% | 15% | 5% |
| 25.4 | Weapons and ammunition | 100% | 20% | 10% |
| 25OTHER | Fabricated metal products, excl. machinery and equipment and weapons & ammunition - 25.1-3/25.5-9 | 100% | 50% | 20% |
| 26 | Computer, electronic and optical products | 100% | 30% | 5% |
| 27 | Electrical equipment | 100% | 35% | 5% |
| 28 | Machinery and equipment n.e.c. | 100% | 15% | 5% |
| 29 | Motor vehicles, trailers and semi-trailers | 28% | 5% | 5% |
| 30.1 | Ships and boats | 100% | 60% | 20% |
| 30.3 | Air and spacecraft and related machinery | 80% | 10% | 5% |
| 30OTHER | Other transport equipment - 30.2/4/9 | 100% | 60% | 20% |
| 31 | Furniture | 50% | 30% | 20% |
| 32 | Other manufactured goods | 97% | 20% | 10% |
| 33.15 | Repair and maintenance of ships and boats | 100% | 70% | 80% |
| 33.16 | Repair and maintenance of aircraft and spacecraft | 100% | 60% | 80% |
| 33OTHER | Rest of repair; Installation - 33.11-14/17/19/20 | 62% | 30% | 80% |
| 35.1 | Electricity, transmission and distribution | 4% | 5% | 5% |
| 35.2-3 | Gas; distribution of gaseous fuels through mains; steam and air conditioning supply | 70% | 40% | 40% |
| 36 | Natural water; water treatment and supply services | 100% | 100% | 100% |
| 37 | Sewerage services; sewage sludge | 68% | 50% | 60% |
| 38 | Waste collection, treatment and disposal services; materials recovery services | 100% | 60% | 100% |
| 39 | Remediation services and other waste management services | 100% | 60% | 100% |
| 41-43 | Construction | 100% | 80% | 90% |
| 45 | Wholesale and retail trade and repair services of motor vehicles and motorcycles | 100% | 70% | 80% |
| 46 | Wholesale trade services, except of motor vehicles and motorcycles | 79% | 40% | 40% |
| 47 | Retail trade services, except of motor vehicles and motorcycles | 100% | 80% | 80% |
| 49.1-2 | Rail transport services | 75% | 50% | 50% |
| 49.3-5 | Land transport services and transport services via pipelines, excluding rail transport | 73% | 40% | 40% |
| 50 | Water transport services | 100% | 40% | 50% |
| 51 | Air transport services | 18% | 10% | 10% |
| 52 | Warehousing and support services for transportation | 35% | 25% | 40% |
| 53 | Postal and courier services | 66% | 50% | 70% |
| 55 | Accommodation services | 100% | 40% | 20% |
| 56 | Food and beverage serving services | 100% | 70% | 80% |
| 58 | Publishing services | 47% | 25% | 15% |
| 59-60 | Motion Picture, Video & TV Programme Production, Sound Recording & Music Publishing services & Programming And Broadcasting services | 18% | 25% | 5% |
| 61 | Telecommunications services | 33% | 30% | 20% |
| 62 | Computer programming, consultancy and related services | 77% | 40% | 80% |
| 63 | Information services | 32% | 20% | 30% |
| 64 | Financial services, except insurance and pension funding | 100% | 30% | 10% |
| 65 | Insurance and reinsurance, except compulsory social security & Pension funding | 62% | 20% | 10% |
| 66 | Services auxiliary to financial services and insurance services | 100% | 30% | 10% |
| 68.1-2 | Real estate services, excluding on a fee or contract basis and imputed rent | 100% | 80% | 80% |
| 68.2IMP | Owner-Occupiers' Housing Services | 100% | 100% | 100% |
| 68.3 | Real estate services on a fee or contract basis | 100% | 90% | 95% |
| 69.1 | Legal services | 55% | 40% | 75% |
| 69.2 | Accounting, bookkeeping and auditing services; tax consulting services | 77% | 60% | 80% |
| 70 | Services of head offices; management consulting services | 57% | 30% | 40% |
| 71 | Architectural and engineering services; technical testing and analysis services | 69% | 60% | 60% |
| 72 | Scientific research and development services | 50% | 25% | 50% |
| 73 | Advertising and market research services | 56% | 30% | 50% |
| 74 | Other professional, scientific and technical services | 92% | 30% | 50% |
| 75 | Veterinary services | 90% | 70% | 70% |
| 77 | Rental and leasing services | 89% | 50% | 70% |
| 78 | Employment services | 73% | 50% | 70% |
| 79 | Travel agency, tour operator and other reservation services and related services | 75% | 30% | 30% |
| 80 | Security and investigation services | 46% | 30% | 70% |
| 81 | Services to buildings and landscape | 64% | 50% | 75% |
| 82 | Office administrative, office support and other business support services | 71% | 60% | 50% |
| 84 | Public administration and defence services; compulsory social security services | 100% | 90% | 90% |
| 85 | Education services | 95% | 60% | 70% |
| 86 | Human health services | 100% | 80% | 80% |
| 87-88 | Residential Care & Social Work services | 100% | 90% | 90% |
| 90 | Creative, arts and entertainment services | 100% | 40% | 50% |
| 91 | Libraries, archives, museums and other cultural services | 100% | 40% | 70% |
| 92 | Gambling and betting services | 63% | 30% | 50% |
| 93 | Sports services and amusement and recreation services | 100% | 70% | 70% |
| 94 | Services furnished by membership organisations | 69% | 60% | 80% |
| 95 | Repair services of computers and personal and household goods | 100% | 40% | 70% |
| 96 | Other personal services | 100% | 90% | 90% |
| 97 | Services of households as employers of domestic personnel | 100% | 100% | 90% |

Table 3 – Values of employment (FTE) forecast under the different scenarios. For details of scenarios, see main text.

| **Sector** | **HAIB** | **LIGB** | **HIGB** | **LIAB** |
| --- | --- | --- | --- | --- |
| Agriculture, forestry and fishing | 15794 | 16282 | 16439 | 16029 |
| Mining activities | 640 | 641 | 642 | 641 |
| Manufacturing | 48463 | 60098 | 63845 | 54064 |
| Electricity and gas | 839 | 853 | 857 | 846 |
| Water and waste | 2801 | 3452 | 3662 | 3114 |
| Construction | 55545 | 66624 | 70192 | 60878 |
| Wholesale and retail | 105579 | 122762 | 128296 | 113850 |
| Transport services | 29085 | 30314 | 30710 | 29676 |
| Accommodation and food services | 45551 | 53557 | 56135 | 49405 |
| Information and communication | 17126 | 17448 | 17551 | 17281 |
| Financial and insurance activities | 23331 | 23550 | 23621 | 23437 |
| Real estate activities | 11432 | 11646 | 11715 | 11535 |
| Professional services | 63013 | 64361 | 64795 | 63662 |
| Administrative and business support services | 41222 | 42642 | 43099 | 41906 |
| Public administration and defence services; compulsory social security services | 25173 | 25360 | 25420 | 25263 |
| Education services | 40757 | 41108 | 41221 | 40926 |
| Health and residential care services | 69510 | 75965 | 78044 | 72617 |
| Creative, arts and entertainment services | 13816 | 14684 | 14963 | 14234 |
| Other services | 20202 | 20706 | 20869 | 20445 |
| Services of households as employers of domestic personnel | 3280 | 3375 | 3406 | 3326 |
